# Clinical exome sequencing: results from 2819 samples reflecting 1000 families

**DOI:** 10.1038/ejhg.2016.146

**Published:** 2016-11-16

**Authors:** Daniel Trujillano, Aida M Bertoli-Avella, Krishna Kumar Kandaswamy, Maximilian ER Weiss, Julia Köster, Anett Marais, Omid Paknia, Rolf Schröder, Jose Maria Garcia-Aznar, Martin Werber, Oliver Brandau, Maria Calvo del Castillo, Caterina Baldi, Karen Wessel, Shivendra Kishore, Nahid Nahavandi, Wafaa Eyaid, Muhammad Talal Al Rifai, Ahmed Al-Rumayyan, Waleed Al-Twaijri, Ali Alothaim, Amal Alhashem, Nouriya Al-Sannaa, Mohammed Al-Balwi, Majid Alfadhel, Arndt Rolfs, Rami Abou Jamra

**Affiliations:** 1 Centogene AG, Rostock, Germany; 2 Division of Genetics, Department of Pediatrics, King Abdulaziz Medical City, Riyadh, Saudi Arabia; 3 College of Medicine, King Saud bin Abdulaziz University for Health Sciences, Riyadh, Saudi Arabia; 4 Division of Neurology, Department of Pediatrics, King Abdulaziz Medical City, Riyadh, Saudi Arabia; 5 Department of pathology, King Abdulaziz Medical City, Riyadh, Saudi Arabia; 6 Division of Metabolic and Genetics, Department of Pediatrics, Prince Sultan Military Medical City, Riyadh, Saudi Arabia; 7 Division of Pediatrics, Johns Hopkins Aramco hospital, Dhahran Health Center, Saudi Aramco, Dhahran, Saudi Arabia; 8 Albrecht-Kossel-Institute for Neuroregeneration, Medical University Rostock, Rostock, Germany; 9 Institute of Human Genetics, University of Leipzig Hospitals and Clinics, Leipzig, Germany

## Abstract

We report our results of 1000 diagnostic WES cases based on 2819 sequenced samples from 54 countries with a wide phenotypic spectrum. Clinical information given by the requesting physicians was translated to HPO terms. WES processes were performed according to standardized settings. We identified the underlying pathogenic or likely pathogenic variants in 307 families (30.7%). In further 253 families (25.3%) a variant of unknown significance, possibly explaining the clinical symptoms of the index patient was identified. WES enabled timely diagnosing of genetic diseases, validation of causality of specific genetic disorders of *PTPN23*, *KCTD3*, *SCN3A*, *PPOX*, *FRMPD4,* and *SCN1B*, and setting dual diagnoses by detecting two causative variants in distinct genes in the same patient. We observed a better diagnostic yield in consanguineous families, in severe and in syndromic phenotypes. Our results suggest that WES has a better yield in patients that present with several symptoms, rather than an isolated abnormality. We also validate the clinical benefit of WES as an effective diagnostic tool, particularly in nonspecific or heterogeneous phenotypes. We recommend WES as a first-line diagnostic in all cases without a clear differential diagnosis, to facilitate personal medical care.

## Introduction

Rare disorders affect around 8% of the world population.^1^ To date, about 7000 different rare diseases are known and a substantially higher number of undefined phenotypes is presumed.^2^ Essentially, 80% of rare diseases are of monogenic origin, and constitute a lifelong risk and a significant burden for the health systems.^3^ Despite high standards in genetic clinics, half of the patients who receive conventional clinical evaluation and targeted genetic testing remain without specific diagnosis even after extensive workup.^4^ This has serious consequences for the patients and their families, preventing the access to the right treatment or accurate counseling for pregnancies and prognosis. Hence, there is growing interest in implementing next generation sequencing (NGS, also called massive parallel sequencing) approaches that deliver fast, and detailed genetic information, providing an effective approach for identifying causal variants in Mendelian disease genes.

Whole exome sequencing (WES), focusing on the most informative regions of the genome, and scanning thousands of genes simultaneously, is an alternative to gene-panel testing and locus specific analysis to investigate the molecular basis of genetic disorders in research and clinical diagnostics set-ups. Published studies about the implementation of WES as a diagnostic tool have been mostly restricted to specific inbred populations or to particular highly selected groups of patients with homogeneous disease presentations.^5, 6, 7, 8, 9, 10^ Here, we present the experience of 1000 consecutive WES requests in our diagnostic clinical routine setup, and validate the use of WES as a first-line diagnostics tool option for patients with a wide range of differential diagnoses or uncharacterized genetic diseases, both in inbred and outbred populations. This comprehensive study includes a highly heterogeneous cohort of 2819 samples from 1000 families referred to us for clinical WES (CentoXome), originating from 54 countries. We demonstrate the high diagnostic value of WES even in a clinically and ethnically heterogeneous cohort, present diagnostic yield in relation to phenotype and family structure information, and we validate recently described genes as causative for specific disorders.

## Materials and methods

### Patient description and ethical considerations

1000 consecutive, unrelated patients referred from physicians from 54 countries of different continents have been included in this study. All patients were referred for diagnostic WES for the period between January 2014 and January 2016 with suspected Mendelian disorders. All analyses were performed in concordance to the provisions of the German Gene Diagnostic Act (Gendiagnostikgesetz) and the General Data Protection Act (Bundesdatenschutzgesetz) to guarantee the confidentiality and protection of data. Written informed consent was obtained of patients or guardians explaining benefits and risks of clinical WES testing. This study was approved by the Ethical Commission of the faculty of Medicine of the University of Rostock (registry no. A 2015-0102). All samples were processed in Centogene's laboratory, which is CAP and CLIA certified, adhering to the ACMG guidelines. Patients and/or their guardians were advised of the potential disclosure of medically actionable incidental findings, and they were given the option of receiving or not such results. Clinical information of all cases was examined by medical experts and human geneticists. We categorized patients' phenotypes according to the Human Phenotype Ontology (HPO) nomenclature^11^ based on the clinical data and preceding workup provided by the referring physician. When available, patient relatives were processed using the same diagnostics workflow (described below) as the index cases.

### DNA extraction

DNA was extracted from EDTA blood or from dry blood spots in filter cards (CentoCard). We used two automated procedures; the spin-column based extraction was performed on QIAcube instrument with QIAamp DNA Blood Mini QIAcube Kit (Qiagen, Valencia, CA, USA) following the manufacturer instructions. Alternatively, the QIAsymphony DSP DNA Mini Kit (Qiagen) on the QIAsymphony instrument was used to purify the DNA from blood. Following extraction all DNA samples were stored at −20 °C. Before the analysis the DNA quality and concentration was determined photometrically (OD260/OD280 1.8–2.0).

### IonTorrent WES workflow

For 911 samples the target regions in the exome were amplified using the Ion AmpliSeq Exome RDY Kit (Life Technologies, Carlsbad, CA, USA). It consists of twelve primer pools (294 000 amplicons) which target >97% of the coding region, and account for a total of 33 Mb. All barcoded samples were sequenced on the Ion Proton with Ion PI Chips v2 taking two samples on a single chip per sequencing run. Sample preparation and chip loading procedure were performed according to the user guide on Ion PI Sequencing 200 Kit v3. Raw sequence data analysis, including base calling, de-multiplexing, alignment to the hg19 human reference genome (Genome Reference Consortium GRCh37), and variant calling, were performed using the Torrent Suite Software v.4.0.2 (Life Technologies).

### Illumina WES workflow

For 1908 samples the exome capture was carried out with Illumina's Nextera Rapid Capture Exome Kit (Illumina, Inc., San Diego, CA, USA). It covers 214 405 exons with a total size of about 37 Mb. Sequencing was done using either the NextSeq500 or HiSeq4000 sequencers (Illumina, Inc.) to produce 2 × 150 bp reads, and always pooling up to 9 WES per lane. Raw sequencing reads were converted to standard fastq format using bcl2fastq software 2.17.1.14 (Illumina, Inc.), and fed to an in-house developed pipeline for the analysis of WES data that is based on the 1000 Genomes Project (1000G) data analysis pipeline and GATK best practice recommendations, which includes widely used open source software projects. The short-reads were aligned to the GRCh37 (hg19) build of the human reference genome using bwa software with the mem algorithm. The alignments were converted to binary bam file format, sorted on the fly, and de-duplicated. The primary alignment files for each sample were further refined and augmented by additional information following GATK best practices recommendations. Afterwards variant calling was performed on the secondary alignment files using three different variant callers (GATK HaplotypeCaller, freebayes, and samtools). A full description of this bioinformatics pipeline can be found in the Supplementary Methods.

### Variant annotation and filtering

Coverage analyses evaluated in a two-step-process the coverage on the single-base level for the complete design and provided detailed statistics on the average coverage as well the percentage of bases with minimum coverage. The RefSeq coding bases and splice junctions considered confidently callable were determined by a minimum of × 10 coverage and no more than 10% MAPQ0 (ambiguously mapped) reads. A × 100 mean depth of coverage was aimed for all samples. Variants were annotated using Annovar^12^ and in-house *ad hoc* bioinformatics tools. Alignments were visually verified with the Integrative Genomics Viewer v.2.3^13^ and Alamut v.2.4.5 (Interactive Biosoftware, Rouen, France). Variant prioritization was performed according to standard procedures with a cascade of filtering steps previously described.^14^ First, all detected variants were initially compared with our internal mutation database (CentoMD), The Human Gene Mutation Database (HGMD), and ClinVar^15^ to directly identify changes previously described in the literature as definitely or likely pathogenic, uncertain, and benign variants. Then, we considered all candidate variants that were identified on both sequenced DNA strands and that account for ≥20% of total reads at that site with a minimum depth of coverage of × 10. Common variants (≥1% in the general population) were discarded by comparison with the 1000G (January 2016, http://www.1000genomes.org), the Exome Variant Server (January 2016, http://evs.gs.washington.edu), the Exome Aggregation Consortium database (ExAC, January 2016, http://exac.broadinstitute.org), and CentoMD (January 2016, http://www.centomd.com), to filter out both common benign variants and recurrent artifact variant calls.

### Evaluation of the pathogenicity of the variants and reporting

All identified variants were considered a priori as variants of unknown significance (VUS). All variants that probably lead to a premature truncated protein (nonsense, frameshifts, affecting initiation codon, single exon, or multi-exon deletions), and all other larger genomic rearrangements, as well as canonical splice site variants (±2 bps) were given high priority. Missense variants and in-frame deletions were evaluated taking into consideration the biophysical and biochemical difference between wild type and changed amino acid, the evolutionary conservation of the nucleotide and amino acid residue in orthologs,^16^ a number of *in silico* predictors (SIFT, Polyphen-2, Mutationtaster among others), and population frequency data. Putative splicing variants were analyzed using Alamut version 2.4.5 (Interactive Biosoftware), a software package that uses different splice site prediction programs to compare the normal and variant sequences for differences in potential regulatory signals. Then, prioritized variants were evaluated based on the suspected disease mode of inheritance and compatibility with the clinical phenotype provided for the index based on several databases and sources of information such as the Online Mendelian Inheritance in Man (OMIM, January 2016, http://omim.org/), HGMD, CentoMD, as well as scientific literature searches in PubMed (http://www.ncbi.nlm.nih.gov/). All clinical features provided were used for each individual case, and, in addition, the HPO ontology was implemented to classify the patient phenotypes. The selected variants were re-evaluated by at least one trained and one senior human geneticist to identify those relevant to the patient's phenotype. Selected candidate variants were classified as pathogenic, likely pathogenic, and VUS according to the criteria published by Richards *et al*,^17^ and were sent for confirmation by conventional PCR amplification and Sanger sequencing. Segregation of these variants with the disease was assessed for all available family members. For reporting, variants were ranked in two main levels according to phenotype compatibility as variants fully or partially explaining the clinical phenotype of the index.

All identified variants in this study have been submitted to the Leiden Open Variation Database 3.0 shared installation (LOVD, http://databases.lovd.nl/shared/, patient IDs 00080793-00081099), and are also available in CentoMD (http://www.centomd.com).

## Results

### Patient demographics and indications for clinical WES referral

We received 1000 index cases for clinical WES diagnostics from 54 different countries, with the largest proportion of patients coming from the Middle East (78.5%), followed by patients coming from Europe (10.6%), and from rest of the world (10.9% Table 1). There were comparable numbers of males and females (1.16:1). Age of index patients ranged between 1 month and 59 years. 14.1% of the index cases were younger than 1 year and the largest age group was from 1–5 years of age (39.4%). Our patient cohort also included 23 prenatal cases (2.3%, Table 1). Most cases (82.7%) were analyzed with a trio design (parents and index), allowing analyses consistent with all possible modes of inheritance of the disease. In 3.4% of the cases one parent was available, in 8.2% none of the parents was available, and in 5.7% other family members were available. Notably, 45.3% of the cases were from consanguineous families (as given in the clinical information), and 38.1% presented family history of the disease.

We applied the HPO system to classify the clinical indications for WES. A summary of the 19 major categories is shown in Table 2. The majority of the patients had an abnormality of the nervous system (*n*=771, 77.1%) with global developmental delay, seizures, and brain malformations as the most common indications. A total of 454 patients (45.4%) presented with abnormalities of head and neck. Facial dysmorphism, microcephaly, and macrocephaly were the main alterations in this group. In addition, 427 cases (42.7%) had abnormality of the musculature, mainly muscular hypotonia/weakness. Growth abnormality (25.2%, mostly failure to thrive), abnormality of the eye (22.6% eg, cataracts and optic atrophy), and abnormality of the metabolism/homeostasis (24.2%, mainly lactic acidosis) were other major categories. Supplementary Table S3 presents a complete list of symptoms of all positive cases. The best diagnostic yield of over 40% was achieved in cases with abnormalities of the connective tissue, of the eye, of the respiratory system, or of metabolism/homeostasis. On the opposite, the diagnostic yield was the lowest in cases with abnormality of prenatal development or birth, of the endocrine system, or of the immune system.

### Variants detected with clinical WES

On the basis of the ACMG classification as described in detail by Richards *et al*,^17^ we identified 320 pathogenic (P) or likely pathogenic (LP) variants accounting for 303 unique variants across 307 of the 1000 cases. The majority of these (*n*=229, 75.6%) were SNVs (107 missense, 79 nonsense, 3 stoploss, and 40 affecting splicing), the rest were small (*n*=72, 23.8%) or large InDels, which were confirmed by MLPA (*n*=2, 0.7%). Most of the identified variants were non-truncating, comprising missense variants or in-frame deletions (*n*=115, 37.9%). Remarkably, about 59.7% (*n*=181) of the pathogenic or likely pathogenic variants were not previously described in any public database (Supplementary Table S1). Here, we do not include variants identified in genes that are not yet confirmed to be associated to human diseases since this goes beyond the diagnostic setting.

### WES diagnostics yield

Overall, 307 out of the 1000 patients undergoing clinical WES had a positive gene finding (30.7%). A total of 165 out of 1000 cases (16.5%) received a definitive molecular diagnosis and in the rest (14.2%) we identified a likely pathogenic variant, thus making the report positive and useful for the family. The majority of the positive cases (*n*=220, 72.6%) had an autosomal recessive disease, followed by patients with an autosomal dominant disease (*n*=70, 23.1%), of these 64 were *de novo* variants (Supplementary Table S1). The remaining cases had X-linked disease (*n*=12, 3.9%). Further, we identified in 253 patients (25.3%) variants of uncertain significance in clinically relevant genes based on OMIM or recent publication in PubMed. However, these were not considered in the estimation of the diagnostic yield. In 44% (*n*=440) of all patients we reported no relevant variant. The presence of consanguinity was linked to higher clinical sensitivity; 34.8% (158/453) in consanguineous families *vs* 27.1% (120/443) in non-consanguineous families, for 104 families we received no information on consanguinity.

### Phenotype complexity and diagnostic yield

**Figure 1:**
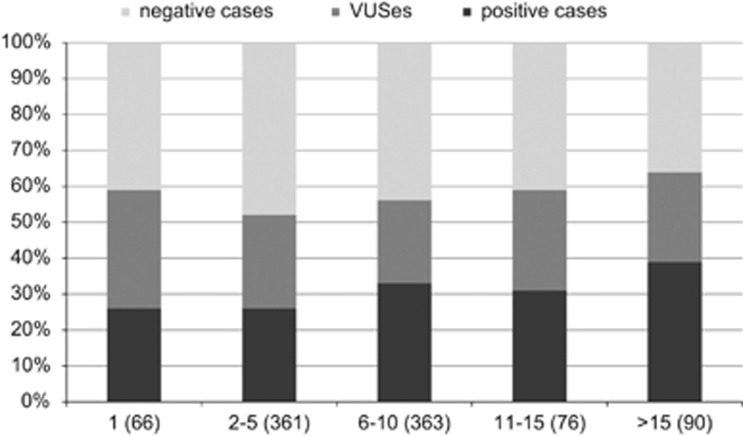
This diagram presents the percentages of cases with a pathogenic/likely pathogenic variant, with a VUS, and that are negative in relation to the complexity of the phenotype represented by the number of HPO terms.

We observed a relation between the complexity of a phenotype of a patient (reflected by the number of HPO terms) and the expected diagnostic yield (Figure 1). If for a case only one HPO term is given, the diagnostic yield is 26%, and stays the same if 2 to 5 HPO terms are given. However, for more complex phenotypes with 6–15 HPO terms the diagnostic yield is remarkably higher (33%) and gets as much as 39% in cases with over 15 HPO terms. Although not statistically significant, this trend applies for all phenotypes and for all examined family structures and inheritance patterns (data not shown). As an example; isolated microcephaly or with up to four additional symptoms has a diagnostic yield of 25%, but this gets to be 42% if there are five or more additional symptoms. Also, we have observed that having a minimum of clinical information, that is, only one single symptom, reduces the specificity of the results, reflected by a high number of reported VUSes.

### Important aspects of the diagnostic yield

In the above mentioned positive 307 cases, a total of 252 genetic diseases were diagnosed with pathogenic or likely pathogenic variants in 247 genes (Supplementary Table S3). Importantly, several of these diagnoses included potentially treatable genetic diseases, with significant implications for patients and their families. Selected examples are cases with ethylmalonic encephalopathy (*ETHE1*), Niemann–Pick disease type C2 (*NPC2*), and pyruvate dehydrogenase E1-alpha deficiency (*PDHA1*). Another important group refers to several metabolic disorders that had remained undiagnosed until WES was performed, although testing for biomarkers, possibly as a screening procedure, would have revealed the diagnosis. Examples are galactosemia (*GALT*), propionic acidemia (*PCCA*), and homocystinuria (*CBS*). In addition, genetic diagnosis was clarified for patients having relatively common and well known genetic disorders such as cystic fibrosis, polycystic kidney disease, and long QT syndrome, suggesting that atypical presentations and highly heterogeneous disorders can eventually be identified by WES. Recurrent pathogenic or likely pathogenic variants in 44 genes were found in two or more unrelated patients (Supplementary Table S2). As an impressive example we have identified in seven patients from five (seemingly) unrelated families in different geographical areas in the Middle East the same variant (c.1A>G; p.(Met1?)) in *C12orf57*, and thus diagnosed Temtamy syndrome.

Noteworthy, among the 307 patients with a positive or likely positive finding, 3 patients received a dual molecular genetic diagnosis. In these patients, two pathogenic or likely pathogenic genetic variants were associated with either non-overlapping clinical presentations or contributing to one major phenotype, that is, the identified pathogenic variants were non-incidental (Table 4).

### Validating novel genes

Finally, we identified variants in genes that were still not in OMIM, but described only rarely, or even only once in a publication (based on PubMed), and were thus able to validate this gene as causative for a specific phenotype (Table 3). These include (a) validating *PTPN23* as a gene for autosomal recessive brain atrophy and developmental delay,^18^ (b) validating *KCTD3* as a gene for autosomal recessive severe intellectual disability and seizures,^18^ (c) validating *SCN3A* as a gene for autosomal dominant encephalopathy,^19, 20^ (d) validating *PPOX* as a gene for autosomal recessive variegate porphyria with developmental delay,^21^ (e) further supporting evidence for the contradictory discussed *FRMPD4* association with X-linked intellectual disability,^22, 23^ and (f) validating the recessive form of Dravet syndrome due to likely pathogenic variants in *SCN1B*.^24^

A full list of all identified pathogenic and likely pathogenic variants can be found in Supplementary Table S3.

## Discussion

Rare diseases caused by altered gene functions are frequently related to severe phenotypes. Yet, if early diagnosis is provided, many patients will have improved life quality or even benefit from medical treatment. Classical genetic diagnosis based on single gene sequencing often lead to negative or inconclusive results.^25, 26^ Clinical WES is a promising diagnostic tool in the routine genetic testing process. To date, reported WES detection rates for deleterious variants in rare disorders encompass 25–30%, but mostly focusing on highly homogenous (geographically or clinically) patient groups.^5, 6, 7, 8, 9, 27, 28, 29^ Other studies that used different classification criteria and highly selected phenotypes or populations reported higher numbers, however not adhering the ACMG guidelines that are needed in a clinical setting.^10, 30^

Here we report the analysis of 1000 consecutive diagnostic cases in a heterogeneous cohort regarding disorder, ethnicity, and family structure/inheritance pattern. Altogether, we reported pathogenic or likely pathogenic variants in 307 of the 1000 families; an overall molecular diagnostic yield of 30.7%. This is considerably higher than the diagnostic rate of standard genetic tests such as chromosomal microarrays, karyotype studies, or single gene test,^31, 32^ especially when taken into account that WES was often not the first choice since genetic testing for differential diagnoses is often performed in advance. The higher diagnostic yield reflects both improvements in technology and in the medical literature. Regarding the latter, we have observed in the presented cohort higher yield in late samples in comparison with the beginnings, and we have re-evaluated negative cases, and could in several cases identify the causative variant or at least a convincing VUS based on literature that has been published in the interim time. Here, we have profited from having all variants of all cases in one database (CentoMD), which enabled re-evaluation on regular basis. An improved diagnostic rate in future analysis due to constant expansions of existing databases of gene-phenotype relations and periodic novel literature entries is imminent.^33^

Out of the total 1000 patients, 440 remained without any relevant variants of clear evidence for pathogenicity and causality. Notwithstanding, additional analysis of family members, further information from referring physicians and reducing technical limitations, and increasing number of gene-phenotype correlations in the literature, will very likely lead to numerous additional positive variant identifications.^34^ Also, recent data on whole genome sequencing (WGS) achievements in genetic diagnosis of human diseases suggest that implementation and widely use of WGS is warranted to offer a better analytical sensitivity.^35, 36^

The mode of inheritance of the reported families in our cohort differs from other reports, with the majority of our positive cases displaying an autosomal recessive mode of inheritance (72.6%). This is very likely due to the enrichment for consanguineous families in our cohort. As anticipated, consanguinity predicted a higher diagnostics yield.^10^

We detected (likely) pathogenic variants in 44 genes, equivalent to 17.8% of total reported genes, present in at least two unrelated patients of our sample collection (Supplementary Table S2). The occurrence of the same homozygous pathogenic variant (c.1A>G p.(Met1?)) in the *C12orf57* gene in five unrelated families, probably due to a founder effect in the Middle East, was an unexpected but most interesting result regarding a potential regional prevalence of a genetic variant. The described variant is associated with Temtamy syndrome, a multiple congenital anomaly syndrome characterized by variable craniofacial dysmorphism, ocular coloboma, seizures, and brain abnormalities. This represents the largest collection of patients with this syndrome due to a single homozygous pathogenic variant. Clinicians from the region are now alerted to suspect this diagnosis in patients presenting with overlapping symptoms.

The fluctuation in the positive reports depending on the requesting institution was between 31 and 44%. However, it seems that this is more dependent on the structure of the families, not on the clinical work out in advance; the clarifying rate of 44% of the institution was in a sample with 71% consanguinity, whereas the clarification rate of 31% was of an institution with 13% consanguinity. Also, there were no significant difference rates of positive reports between different regions in the world, especially when considering the different rates of consanguinity; for example, Europe 32% and Middle East 36%.

We also have observed that complex phenotypes, that is, which include several symptoms, tend to have a higher diagnostic yield (Figure 1). We still cannot give a final explanation for this observation. However, the following factors may have a role: (a) having one or only few symptoms extend the number of candidate genes with overlapping symptoms, thus making specifying one single variant more difficult, which is reflected by the high number of VUSes in such cases; (b) phenotype with several symptoms have a higher probability of being due to a monogenic reason, in opposite to patients with single symptom (eg, diarrhea, failure to thrive, polyhydramnios, autism, or parkinsonism) that may be due to a genetic complex etiology; (c) complex phenotypes are better studied in the literature than phenotypes with one single symptom; and (d) giving several symptoms has a higher probability of including the qualitatively decisive symptom. Thus, we recommend physicians who request a WES to include as many symptoms as possible in their clinical description, but to mark those that seem to be specific for this patient. We recommend for WES evaluation to consider rather less detailed clinical description by restricting this to around 3–10 leading and/or specific symptoms.

To establish a justified clinical diagnosis from WES results, the requesting medical geneticists and the diagnostics laboratory need to be concordant in their interpretation of these results.^37^ The overall strategy of our clinical WES workflow is a very intensive collaboration with clinicians before, during, and after WES analysis to provide relevant molecular findings. The essential benefit of close cooperation not only lies in the identification of formerly unknown diseases caused by rare genetic variants, as shown in a separate case reports of our results on novel asparagine synthetase deficiency and skeletal ciliopathy,^38, 39^ but also allowing future evidence based evaluation by providing combined genetic and clinical information.

For the VUSes that we have identified, retrospective clinical examining of the patients may enable a better evaluation by confirming relevance, or by excluding the identified variant.

We identified pathogenic or likely pathogenic variants in two different genes in three patients (Table 4). This is a particular feature of WES, demonstrating its advantage compared with traditional diagnostic methods, especially when dealing with complex phenotypes. The delivery of dual diagnosis would certainly not be as straight forward with current classical genetic tools applied in clinical diagnostics.

Other clear diagnostic advantage of WES is the possibility of fast validation of genes described to be associated with a genetic disease by a single publication, which thus do not find access to OMIM. Our findings validate the link between variants in six such genes and specific phenotypes (Table 3).

Altogether, our results strongly support diagnostic WES as a first diagnostic choice if there is no clear differential diagnosis. We think that chromosomal analysis and repeat expansion diagnostics would still clarify a significant part of intellectual disability, but we plead for WES as soon as a clear differential diagnosis is not available. Overall the superiority of clinical WES over standard genetic tests is illustrated by the broad simultaneous coverage of thousands of genes, by a low-cost and fast turnaround approach, but also by the unique potential for dual molecular diagnosis and efficient identification of variants across diverse phenotypes and populations. Widespread WES implementation will allow more tailored medical care based on individual risk.

## Figures and Tables

**Table 1:** Geographic origin of the 1000 families and the 307 patients with pathogenic/likely pathogenic variants tested by clinical WES

*Geographic region*	*No. of patients in total (%*^a^)	*No. of patients with P/LP variants (%*^b^)
Middle East	785 (78.5)	244 (79.5)
South / North America	42 (4.2)	8 (2.6)
Europe	106 (10.6)	33 (10.8)
South Asia	58 (5.8)	21 (6.9)
Oceania	8 (0.8)	1 (0.3)
South Africa	1 (0.1)	0 (0)
		
*Age of Patients*		
Prenatal	23 (2.3)	4 (1.3)
<1 year	141 (14.1)	42 (13.7)
1–5 years	394 (39.4)	128 (41.7)
5–15 years	285 (28.5)	73 (23.8)
15–30 years	81 (8.1)	23 (7.5)
>30 years	38 (3.8)	10 (3.3)
Unknown	38 (3.8)	27 (8.8)
Total	1000	307
Consanguineous	453/1000 (45.3)	158/307 (51.5)

Abbreviations: LP, likely pathogenic; P, pathogenic. Age distribution and consanguinity are shown as well.

a % among the 1000 cases.

b % among the 307 positive/likely positive WES cases.

**Table 2:** Motive of clinical WES request among 1000 families categorized according to HPO and their distribution according to genetic findings (with pathogenic or likely pathogenic variants)

*Symptoms class*	*All(%)*	*P/LP (% from all cases)* a	*P/LP (% of* 307 *positive cases)* b	*P/LP (% of patients from same phenotype class)* c
Abnormality of the nervous system	771 (77.1)	229 (22.9)	74.6	29.7
Abnormality of head or neck	454 (45.4)	143 (14.3)	46.6	31.5
Abnormality of the musculature	427 (42.7)	152 (15.2)	49.5	35.6
Abnormality of the skeletal system	398 (39.8)	132 (13.2)	43.0	33.2
Growth abnormality	252 (25.2)	76 (7.6)	24.8	30.2
Abnormality of metabolism/homeostasis	242 (24.2)	86 (8.6)	28.0	35.5
Abnormality of the abdomen	233 (23.3)	76 (7.6)	24.8	32.6
Abnormality of the eye	226 (22.6)	90 (9)	29.3	39.8
Abnormality of the integument	167 (16.7)	60 (6)	19.5	35.9
Abnormality of the cardiovascular system	177 (17.7)	49 (4.9)	15.9	27.7
Abnormality of the genitourinary system	157 (15.7)	50 (5)	16.3	31.8
Abnormality of limbs	137 (13.7)	48 (4.8)	15.6	35.0
Abnormality of the ear	132 (13.2)	41 (4.1)	13.4	31.1
Abnormality of the immune system	106 (10.6)	27 (2.7)	8.8	25.5
Abnormality of the respiratory system	107 (10.7)	41 (4.1)	13.4	38.3
Abnormality of prenatal development or birth	84 (8.4)	22 (2.2)	7.2	26.2
Abnormality of blood and blood-forming tissues	70 (7)	24 (2.4)	7.8	34.3
Abnormality of connective tissue	55 (5.5)	24 (2.4)	7.8	43.6
Abnormality of the endocrine system	43 (4.3)	11 (1.1)	3.6	25.6

Abbreviations: LP, likely pathogenic; P, pathogenic.

a P/LP patients of particular symptoms class in relation to total patients.

b P/LP patients of particular symptoms class in relation to total P/LP patients.

c P/LP patients of particular symptoms class in relation to all patients of this particular symptoms class.

**Table 4:** Patients with dual molecular diagnosis by clinical WES

*LOVD patient ID*	*Inheritance*	*Gene*	*OMIM description*	*OMIM Id*	*Transcript*	*Mutation*	*Type of Mutation*	*Class*	*HPO terms*
00080793	AR	*INSR*	Leprechaunism	OMIM:246200	NM_000208.2	c.433C>T	Missense	P	Lymphedema, Intrauterine growth retardation, hypertrophic cardiomyopathy, cardiomegaly, patent ductus arteriosus, mitral regurgitation, left ventricular hypertrophy, immunodeficiency, decreased skull ossification
	AR	*IFNGR2*	Immunodeficiency 28, mycobacteriosis	OMIM:614889	NM_005534.3	c.705C>A	Nonsense	P	
00080794	AD	*FOXP1*	Mental retardation with language impairment and with or without autistic features	OMIM: 613670	NM_032682.5	c.1573C>T	Nonsense	P	Macrocephaly, abnormality of the face, low-set ears, delayed speech and language development, intellectual disability, motor delay, agenesis of corpus callosum, megalencephaly, postaxial polydactyly
	AD	*PTCH1*	Basal cell nevus syndrome	OMIM: 109400	NM_000264.3	c.2834delinsCGGGTCCACAACATC	Frameshift	LP	
00080795	AR	*DLD*	Dihydrolipoamide dehydrogenase deficiency	OMIM:246900	NM_000108.3	c.685G>T	Missense	P	Abnormality of coagulation, hypoglycemia, vomiting, hyperuricemia, hepatomegaly, elevated hepatic transaminases, lactic acidosis, decreased muscle mass, fatigable weakness, abnormal eating behavior
	Mitochondrial	*MT-CO1*	Cytochrome c oxidase subunit I	OMIM:516030	NC_012920.1	m.7443A>C	Stoploss	LP	

Abbreviations: AD, autosomal dominant; AR, autosomal recessive; LP, likely pathogenic; P, pathogenic.

**Table 3:** Validation of recently reported genes as associated with specific disorders

*Gene*	*Transcript*	*Inheritance, zygosity*	*cDNA change*	*AA change*	*Family segregation*	*Significance*	*Symptoms of patient (in HPO terms)*
*KCTD3*	NM_016121.3	AR, homozygous	c.1036_1073del	p.(P346Tfs*4)	Inherited from parents	Likely Pathogenic	Hydrocephalus, delayed speech and language development, seizures, global developmental delay, Dandy–Walker malformation, polymicrogyria, abnormality of the cerebral white matter, abnormal cortical gyration
*PPOX*	NM_000309.3	AR, homozygous	c.1108_1119del	p.(G370_W373del)	Inherited from parents	Likely Pathogenic	Nystagmus, hypopigmentation of the skin, seizures, leukodystrophy, ichthyosis, primary adrenal insufficiency, abnormality of the heme biosynthetic pathway, neonatal asphyxia, inappropriate crying
*SCN1B*	NM_001037.4	AR, homozygous	c.449-2A>G	—	Inherited from parents	Likely pathogenic	Global developmental delay, hyperreflexia, generalized myoclonic seizures, muscular hypotonia of the trunk, feeding difficulties, epileptic encephalopathy
*SCN3A*	NM_006922.3	AD, heterozygous	c.3998C>T	p.(P1333L)	*De nov*o	VUS	Seizures, generalized tonic-clonic seizures, febrile seizures, delayed myelination
*PTPN23*	NM_015466.2	AR, homozygous	c.904A>G	p.(M302V)	Inherited from parents	VUS	Microcephaly, delayed speech and language development, seizures, spasticity,global developmental delay, motor delay, developmental regression, brain atrophy, abnormality of movement
*FRMPD4*	NM_014728.3	AR, homozygous	c.380C>T	p.(P127L)	Inherited from parents	VUS	Intellectual disability
