# Supplementary material for: Clinical exome sequencing: results from 2819 samples reflecting 1000 families
Source: Eur J Hum Genet. 2016 Nov 16;25(2):176–82. doi: 10.1038/ejhg.2016.146 (PMC5255946; doi:10.1038/ejhg.2016.146)
Supplement: Supplementary Table 1 [file ejhg2016146x1.docx]

| **Table S1.** Distribution of the identified pathogenic and likely pathogenic variants according to disease pattern of inheritance | | | |  |  |
| --- | --- | --- | --- | --- | --- |
|  |  |  |  |  |  |
|  | **Autosomal Dominant Allele(%^a^)** | **Autosomal Recessive Allele(%^a^)** | **X-linked Allele(%^a^)** | **Mitochondrial** | **Total(%^a^)** |
| **Missense** | 33(10.89) | 70(23.1) | 4(1.32) | 0(0) | 107(35.31) |
| **Nonsense** | 18(5.94) | 56(18.48) | 5(1.65) | 0(0) | 79(26.07) |
| **Stoploss** | 0(0) | 2(0.66) | 0(0) | 1(0.33) | 3(0.99) |
| **Splicing** | 5(1.65) | 34(11.22) | 1(0.33) | 0(0) | 40(13.2) |
| **Small frameshift** | 14(4.62) | 48(15.84) | 2(0.66) | 0(0) | 64(21.12) |
| **In-Frame deletion** | 0(0) | 8(2.64) | 0(0) | 0(0) | 8(2.64) |
| **Large deletion** | 0(0) | 2(0.66) | 0(0) | 0(0) | 2(0.66) |
| **Novel variant(out of 303 L/LP variants)** | 54(17.82) | 119(39.27) | 7(2.31) | 1(0.33) | 181(59.74) |
|  |  |  |  |  |  |
|  |  |  |  |  |  |
| ^a^% among the 303 pathogenic/likely pathogenic variants, P-Pathogenic, LP-Likely pathogenic | | |  |  |  |
