# Supplementary material for: Clinical exome sequencing: results from 2819 samples reflecting 1000 families
Source: Eur J Hum Genet. 2016 Nov 16;25(2):176–82. doi: 10.1038/ejhg.2016.146 (PMC5255946; doi:10.1038/ejhg.2016.146)
Supplement: Supplementary Table 2 [file ejhg2016146x2.docx]

| **Table S2.** Recurrent genetic findings among cases. | | |  | |  | | |  | |
| --- | --- | --- | --- | --- | --- | --- | --- | --- | --- |
| **Gene** | **Occurrence^a^** | **OMIM description_new/Pubmed Description** | | **OMIM** | | **No of variants ^b^** | **Pathogenic** | | **Likely pathogenic** |
| *PLA2G6* | 7 | Infantile neuroaxonal dystrophy 1/Neurodegeneration with brain iron accumulation 2B | | OMIM:256600/OMIM:610217 | | 5 | 4 | | 1 |
| *C12orf57* | 5 | Temtamy syndrome | | OMIM:218340 | | 1 | 1 | | 0 |
| *FBXL4* | 4 | Mitochondrial DNA depletion syndrome 13 (encephalomyopathic type) | | OMIM:615471 | | 3 | 0 | | 3 |
| *ADCK3* | 4 | Coenzyme Q10 deficiency, primary, 4 | | OMIM:612016 | | 4 | 2 | | 2 |
| *CLN6* | 3 | Ceroid lipofuscinosis, neuronal, 6 | | OMIM:601780 | | 3 | 1 | | 2 |
| *ADAT3* | 3 | Mental retardation, autosomal recessive 36 | | OMIM:615286 | | 1 | 1 | | 0 |
| *COLQ* | 3 | Myasthenic syndrome, congenital, 5 | | OMIM:603034 | | 3 | 2 | | 1 |
| *DLD* | 3 | Dihydrolipoamide dehydrogenase deficiency | | OMIM:246900 | | 2 | 2 | | 0 |
| *FKRP* | 3 | Muscular dystrophy-dystroglycanopathy (congenital with or without mental retardation), type B, 5 | | OMIM:606612 | | 3 | 3 | | 0 |
| *KMT2A* | 3 | Wiedemann-Steiner syndrome | | OMIM:605130 | | 3 | 0 | | 3 |
| *IGHMBP2* | 3 | Charcot-Marie-Tooth disease, axonal, type 2S /Neuronopathy, distal hereditary motor, type VI | | OMIM:616155/OMIM:604320 | | 3 | 3 | | 0 |
| *WWOX* | 3 | Epileptic encephalopathy, early infantile, 28, Autosomal recessive | | OMIM:616211 | | 3 | 0 | | 3 |
| *SNX14* | 3 | Spinocerebellar ataxia, autosomal recessive 20 | | OMIM:616354 | | 2 | 1 | | 1 |
| *GRIN2B* | 2 | Mental retardation, autosomal dominant 6/Epileptic encephalopathy, early infantile, 27 | | OMIM:613970/OMIM:616139 | | 2 | 0 | | 2 |
| *KCNQ2* | 2 | Epileptic encephalopathy, early infantile, 7/Seizures, benign neonatal, 1 | | OMIM:613720/OMIM:121200 | | 2 | 1 | | 1 |
| *ALDH7A1* | 2 | Epilepsy, pyridoxine-dependent | | OMIM:266100 | | 2 | 1 | | 1 |
| *ASNS* | 2 | Asparagine synthetase deficiency | | OMIM:615574 | | 1 | 1 | | 0 |
| *ASPM* | 2 | Microcephaly 5, primary, autosomal recessive | | OMIM:608716 | | 2 | 1 | | 1 |
| *ADK* | 2 | Hypermethioninemia due to adenosine kinase deficiency | | OMIM:614300 | | 2 | 0 | | 2 |
| *ARID1B* | 2 | Mental retardation, autosomal dominant 12 | | OMIM:614562 | | 2 | 0 | | 2 |
| *CA2* | 2 | Osteopetrosis, autosomal recessive 3, with renal tubular acidosis | | OMIM:259730 | | 2 | 1 | | 1 |
| *CDKL5* | 2 | Epileptic encephalopathy, early infantile, 2 | | OMIM:300672 | | 2 | 0 | | 2 |
| *CLCNKB* | 2 | Bartter syndrome type 4B | | OMIM:613090 | | 2 | 2 | | 0 |
| *COL6A2* | 2 | Ullrich congenital muscular dystrophy 1 | | OMIM:254090 | | 1 | 0 | | 1 |
| *CTNNB1* | 2 | Mental retardation, autosomal dominant 19 | | OMIM:615075 | | 2 | 0 | | 2 |
| *DGUOK* | 2 | Mitochondrial DNA depletion syndrome 3 (hepatocerebral type) | | OMIM:251880 | | 2 | 2 | | 0 |
| *HEXB* | 2 | Sandhoff disease, infantile, juvenile, and adult forms | | OMIM:268800 | | 2 | 2 | | 0 |
| *HRAS* | 2 | Costello syndrome | | OMIM:218040 | | 2 | 2 | | 0 |
| *INSR* | 2 | Leprechaunism/Rabson-Mendenhall syndrome | | OMIM:246200/OMIM:262190 | | 1 | 1 | | 0 |
| *KCNQ1* | 2 | Jervell and Lange-Nielsen syndrome | | OMIM:220400 | | 2 | 2 | | 0 |
| *L2HGDH* | 2 | L-2-hydroxyglutaric aciduria | | OMIM:236792 | | 2 | 1 | | 1 |
| *MECP2* | 2 | Rett syndrome | | OMIM:312750 | | 1 | 1 | | 0 |
| *MFSD8* | 2 | Ceroid lipofuscinosis, neuronal, 7 | | OMIM:610951 | | 2 | 0 | | 2 |
| *MYO7A* | 2 | Usher syndrome, type 1B | | OMIM:276900 | | 2 | 2 | | 0 |
| *NF1* | 2 | Neurofibromatosis type 1 | | OMIM:162200 | | 2 | 1 | | 1 |
| *NSD1* | 2 | Sotos syndrome 1 | | OMIM:117550 | | 2 | 2 | | 0 |
| *PSAP* | 2 | Combined SAP deficiency | | OMIM:611721 | | 1 | 1 | | 0 |
| *RAB3GAP1* | 2 | Warburg micro syndrome 1 | | OMIM:600118 | | 2 | 1 | | 1 |
| *SGCA* | 2 | Muscular dystrophy, limb-girdle, type 2D | | OMIM:608099 | | 1 | 1 | | 0 |
| *SLC13A5* | 2 | Epileptic encephalopathy, early infantile, 25 | | OMIM:615905 | | 2 | 1 | | 1 |
| *STXBP1* | 2 | Epileptic encephalopathy, early infantile, 4 | | OMIM:612164 | | 2 | 2 | | 0 |
| *TJP2* | 2 | Cholestasis, progressive familial intrahepatic 4 | | OMIM:615878 | | 1 | 0 | | 1 |
| *TTN* | 2 | Myopathy, early-onset, with fatal cardiomyopathy | | OMIM:611705 | | 2 | 0 | | 2 |
| *WDR81* | 2 | Cerebellar ataxia, mental retardation, and dysequilibrium syndrome 2 | | OMIM:610185 | | 2 | 0 | | 2 |

^a^ Total number of occurrences of a gene variant in different families, ^b^ Total number of different variants in the same gene
