# Supplementary material for: Clinical exome sequencing: results from 2819 samples reflecting 1000 families
Source: Eur J Hum Genet. 2016 Nov 16;25(2):176–82. doi: 10.1038/ejhg.2016.146 (PMC5255946; doi:10.1038/ejhg.2016.146)
Supplement: Supplementary Table 3 [file ejhg2016146x3.docx]

| **Table S3.** List of variants identified among positive/likely positive results from probands undergoing diagnostic WES. | | | | | | | | | | | | | | | | |
| --- | --- | --- | --- | --- | --- | --- | --- | --- | --- | --- | --- | --- | --- | --- | --- | --- |
| **LOVD patient ID** | **Geographic Region** | **Sex** | **Age (years)** | **Consanguinity** | **Positive Family History** | **Gene** | **Transcript** | **OMIM description_new/Pubmed Description (OMIM/Pubmed Id)** | **Inheritance** | **Zygosity** | **Coding Effect** | **cDNA change** | **AA change** | **Segregation** | **Significance** | **HPO terms** |
| 00080793 | ME | f | 0.4 | Yes | Yes | INSR | NM_000208.2 | Rabson-Mendenhall syndrome (OMIM:262190) | AR | hom | Missense | c.433C>T | p.(R145C) | Inherited from parents | P | Lymphedema,Intrauterine growth retardation,Hypertrophic cardiomyopathy,Cardiomegaly,Patent ductus arteriosus,Mitral regurgitation,Left ventricular hypertrophy,Immunodeficiency,Decreased skull ossification |
|  |  |  |  |  |  | IFNGR2 | NM_005534.3 | Immunodeficiency 28, mycobacteriosis (OMIM:614889) | AR | hom | Nonsense | c.705C>A | p.(Y235*) | Inherited from parents | P |  |
| 00080794 | Eu | f | 7.7 | N.i | N.i | FOXP1 | NM_032682.5 | Mental retardation with language impairment and with or without autistic features (OMIM: 613670) | AD | het | Nonsense | c.1573C>T | p.(R525*) | De Novo | P | Macrocephaly,Abnormality of the face,Low-set ears,Delayed speech and language development,Intellectual disability,Motor delay,Agenesis of corpus callosum,Megalencephaly,Postaxial polydactyly |
|  |  |  |  |  |  | PTCH1 | NM_000264.3 | Basal cell nevus syndrome (OMIM: 109400) | AD | het | Frameshift | c.2834delinsCGGGTCCACAACATC | p.(R945Qfs*22) | De Novo | LP |  |
| 00080795 | ME | f | 5.9 | Yes | No | DLD | NM_000108.3 | Dihydrolipoamide dehydrogenase deficiency (OMIM:246900) | AR | hom | Missense | c.685G>T | p.(G229C) | Inherited from parents | P | Abnormality of coagulation,Hypoglycemia,Vomiting,Hyperuricemia,Hepatomegaly,Elevated hepatic transaminases,Lactic acidosis,Decreased muscle mass,Fatigable weakness,Abnormal eating behavior |
|  |  |  |  |  |  | MT-CO1 | NC_MTCO1_012920.1 | Cytochrome c oxidase subunit I   (OMIM:516030) | M | Heteroplasmic | stoploss | m.7443A>C | p.(*514Rext) | Inherited from mother | LP |  |
| 00080796 | Eu | m | 3.3 | N.i | No | MMAA | NM_172250.2 | Methylmalonic aciduria, vitamin B12-responsive (OMIM:251100) | AR | comp het | Frameshift | c.593_596del | p.(T198Sfs*6) | Inherited from father | P | Autism,Muscular hypotonia,Global developmental delay,Motor delay,Joint hypermobility,Ketosis,Organic aciduria,Vomiting,Constipation,Febrile seizures,Developmental regression |
|  |  |  |  |  |  |  |  |  |  |  | Frameshift | c.651dup | p.(G218Rfs*9) | Inherited from mother | LP |  |
| 00080797 | ME | f | 20.2 | No | N.i | ADCK3 | NM_020247.4 | Coenzyme Q10 deficiency, primary, 4 (OMIM:612016) | AR | comp het | In-frame | c.1750_1752del | p.(T584del) | Inherited from mother | P | Nystagmus,Ataxia,Dystonia,Chorea,Abnormality of movement |
|  |  |  |  |  |  |  |  |  |  |  | Missense | c.1805C>G | p.(P602R) | Inherited from father | P |  |
| 00080798 | Am | f | 45.8 | No | Yes | NEU1 | NM_000434.3 | Sialidosis, type I (OMIM: 256550) | AR | comp het | Missense | c.700G>A | p.(D234N) | Inherited from father | P | Ataxia,Spasticity,Dysarthria,Dysmetria,Hyperreflexia,Gait ataxia,Spastic paraparesis |
|  |  |  |  |  |  |  |  |  |  |  | Frameshift | c.344del | p.(M115Rfs*23) | Inherited from mother | LP |  |
| 00080799 | Eu | m | 2.8 | No | No | WWOX | NM_016373.3 | Epileptic encephalopathy, early infantile, 28, Autosomal recessive (OMIM:616211) | AR | comp het | Splicing | c.173-1G>T | p.? | Inherited from mother | LP | Hearing impairment,Optic atrophy,Delayed speech and language development,Intellectual disability,Seizures,Global developmental delay,Hypertonia,Muscle weakness,Abnormally lax or hyperextensible skin,Brain atrophy |
|  |  |  |  |  |  |  |  |  |  |  | Frameshift | c.918del | p.(E306Dfs*21) | Inherited from father | LP |  |
| 00080800 | ME | m | unknown | N.i | No | ABCA12 | NM_173076.2 | Ichthyosis, autosomal recessive 4B (harlequin) (OMIM:242500) | AR | comp het | Nonsense | c.1866del | p.(C622*) | Inherited from mother | LP | Ichthyosis,Sepsis |
|  |  |  |  |  |  |  |  |  |  |  | Nonsense | c.3666C>A | p.(Y1222*) | Inherited from father | LP |  |
| 00080801 | ME | u | unknown | No | Yes | ACE | NM_000789.3 | Renal tubular dysgenesis (OMIM:267430) | AR | comp het | Nonsense | c.793C>T | p.(R265*) | Inherited from father | P | Renal insufficiency,Renal tubular dysfunction,High palate,Low-set ears,Single transverse palmar crease,Oligohydramnios,Abnormal renal morphology |
|  |  |  |  |  |  |  |  |  |  |  | Splicing | c.1586+1G>A | p.? | Inherited from mother | LP |  |
| 00080802 | ME | f | 0.2 | No | No | AHCY | NM_000687.2 | Hypermethioninemia with deficiency of S-adenosylhomocysteine hydrolase (OMIM:613752) | AR | comp het | Missense | c.266C>T | p.(A89V) | Inherited from father | P | Muscle weakness,Abnormal levels of creatine kinase in blood |
|  |  |  |  |  |  |  |  |  |  |  | Missense | c.428A>G | p.(Y143C) | Inherited from mother | P |  |
| 00080803 | Eu | m | 4.3 | Yes | No | TRAPPC9 | NM_031466.6 | Mental retardation, autosomal recessive 13 (OMIM:613192) | AR | comp het | Nonsense | c.1423C>T | p.(R475*) | Inherited from mother | P | Cryptorchidism,Wide mouth,Abnormality of upper lip,Brachycephaly,Microcephaly,Smooth philtrum,Small forehead,Short neck,Visual impairment,Delayed speech and language development,Abnormal external genitalia,Hypertrichosis,Intellectual disability,Global developmental delay,Motor delay,Agenesis of corpus callosum,Abnormality of the cerebral ventricles,Leukodystrophy,Long eyebrows,Cerebral dysmyelination,Brain atrophy |
|  |  |  |  |  |  |  |  |  |  |  | Splicing | c.3350-2A>G | p.? | Inherited from father | LP |  |
| 00080804 | ME | m | 1.0 | No | Yes | MPV17 | NM_002437.4 | Mitochondrial DNA depletion syndrome 6 (hepatocerebral type ()OMIM:256810) | AR | comp het | Missense | c.148C>T | p.(R50W) | Inherited from mother | P | Lactic acidosis,Fatal liver failure in infancy,Chronic hepatic failure |
|  |  |  |  |  |  |  |  |  |  |  | Frameshift | c.284dup | p.(F96Lfs*17) | Inherited from father | LP |  |
| 00080805 | ME | m | 4.7 | No | No | TPP1 | NM_000391.3 | Ceroid lipofuscinosis, neuronal, 2 (OMIM:204500) | AR | comp het | Missense | c.833A>C | p.(Q278P) | Inherited from mother | LP | Delayed speech and language development,Intellectual disability,Seizures,Ataxia,Motor delay,Developmental regression,Brain atrophy |
|  |  |  |  |  |  |  |  |  |  |  | Nonsense | c.622C>T | p.(R208*) | Inherited from father | P |  |
| 00080806 | ME | f | 1.4 | No | N.i | CLCNKB | NM_000085.4 | Bartter syndrome type 4B(OMIM:613090) | AR | hom | Large Del | c.-152_576del | p.? | N.i | P | Failure to thrive,Abnormality of metabolism/homeostasis,Alkalosis,Hypokalemia |
| 00080807 | ME | f | 3.5 | Yes | Yes | WDR81 | NM_001163809.1 | Cerebellar ataxia, mental retardation, and dysequilibrium syndrome 2 (OMIM:610185) | AR | hom | Nonsense | c.3997C>T | p.(R1333*) | N.i | LP | Thin vermilion border,Abnormality of the face,Facial hemangioma,Conductive hearing impairment,Deeply set eye,Muscular hypotonia,Global developmental delay,Unsteady gait,Paraplegia,Brain atrophy,Delayed myelination |
| 00080808 | SA | m | 3.3 | Yes | Yes | SURF1 | NM_003172.3 | Leigh syndrome, COX IV deficiency(OMIM:256000) | AR | hom | Frameshift | c.19_35del | p.(Leu7Glyfs*47) | Inherited from parents | LP | Delayed speech and language development,Hirsutism,Global developmental delay,Episodic fever,Unsteady gait,Limb tremor |
| 00080809 | ME | f | 13.5 | Yes | No | INSR | NM_000208.2 | Rabson-Mendenhall syndrome(OMIM:262190) | AR | hom | Missense | c.433C>T | p.(R145C) | Inherited from parents | P | Enlarged kidneys,Mandibular prognathia,Hyperinsulinemia,Abnormality of skin pigmentation,Hirsutism,Abnormality of the hair,Abnormal facial shape,Increased circulating ACTH level,Generalized hyperpigmentation,Ichthyosis |
| 00080810 | ME | f | 1.6 | Yes | No | COLQ | NM_005677.3 | Myasthenic syndrome, congenital, 5(OMIM:603034) | AR | hom | Frameshift | c.738del | p.(V247Lfs*29) | Inherited from parents | P | Ptosis,Ophthalmoplegia,Delayed speech and language development,Muscular hypotonia,Motor delay,Weak cry,Dysphagia |
| 00080811 | Eu | m | 1.5 | No | No | COL2A1 | NM_001844.4 | Stickler syndrome, type I (OMIM:108300) | AD | het | Missense | c.926G>T | p.(G309V) | Inherited from father | LP | Abnormality of the skeletal system,Short femur,Short stature,Short tibia,Short lower limbs |
| 00080812 | ME | f | 3.3 | No | No | PLA2G6 | NM_003560.2 | Infantile neuroaxonal dystrophy 1 (OMIM:256600) | AR | hom | Nonsense | c.2370T>G | p.(Y790*) | Inherited from father& | P | Microcephaly,Strabismus,Myopia,Cyanosis,Muscular hypotonia,Motor delay,Muscle weakness,Myopathic facies,Ketonuria,Weakness of the intrinsic hand muscles,Abnormal stereopsis |
| 00080813 | ME | m | 6.3 | No | N.i | PLA2G6 | NM_003560.2 | Infantile neuroaxonal dystrophy 1 (OMIM:256600) | AR | hom | In-frame | c.2070_2072del | p.(V691del) | Inherited from parents | P | Optic atrophy,Spasticity,Leukodystrophy |
| 00080814 | ME | f | 4.7 | Yes | No | PLA2G6 | NM_003560.2 | Infantile neuroaxonal dystrophy 1 (OMIM:256600) | AR | hom | In-frame | c.2070_2072del | p.(V691del) | Inherited from parents | P | Optic atrophy,Muscular hypotonia,Spasticity,Global developmental delay,Leukodystrophy,Brain atrophy |
| 00080815 | Eu | f | 3.1 | Yes | No | PLA2G6 | NM_003560.2 | Infantile neuroaxonal dystrophy 1(OMIM:256600) | AR | hom | Frameshift | c.2370_2371del | p.(Y790*) | Inherited from parents | P | Strabismus,Visual impairment,Hypermetropia,Optic atrophy,Delayed speech and language development,Motor delay,Cerebellar hypoplasia,Developmental regression,Multiple joint contractures,Sleep apnea,Demyelinating sensory neuropathy,Childhood onset sensorineural hearing impairment |
| 00080816 | ME | f | 9.2 | Yes | Yes | PLA2G6 | NM_003560.2 | Infantile neuroaxonal dystrophy type 1(OMIM:256600) | AR | hom | Nonsense | c.2098C>T | p.(Q700*) | Inherited from parents | LP | Strabismus,Visual impairment,Nystagmus,Spasticity,Cerebellar hypoplasia,Vacuolated lymphocytes,Generalized seizures,Developmental regression,Scoliosis,Brain atrophy |
| 00080817 | ME | f | 11.3 | yes | n.i | SNX14 | NM_153816.3 | Spinocerebellar ataxia type 20(OMIM:616354) | AR | hom | stopgain | c.2722C>T | p.(Q908*) | inherited from parents | LP | Sensorineural hearing impairment, Visual impairment, Optic atrophy, Delayed speech and language development, Intellectual disability, Motor delay, Muscle weakness, Abnormality of the hair, Abnormal facial shape, Hip dislocation, Short stature |
| 00080818 | ME | f | 3.5 | n.i | no | AP4E1 | NM_007347.4 | Spastic paraplegia 51(OMIM:613744) | AR | hom | frameshift | c.1036_1037del | p.(L346Vfs*3) | inherited from parents | LP | Delayed speech and language development, Intellectual disability, Seizures, Motor delay, Congenital microcephaly, Brain atrophy |
| 00080819 | Eu | f | 4.3 | no | no | PPP2R5D | NM_006245.3 | Mental retardation, autosomal dominant 35(OMIM:616355) | AD | het | missense | c.592G>A | p.(E198K) | de novo | P | High palate, Macrocephaly, Nystagmus, Delayed speech and language development, Intellectual disability, Muscular hypotonia, Motor delay |
| 00080820 | ME | f | 2.1 | Yes | No | PLA2G6 | NM_003560.2 | Neurodegeneration with brain iron accumulation 2B(OMIM:610217) | AR | hom | In-frame | c.2070_2072del | p.(V691del) | Inherited from mother& | P | Macrocephaly,Irritability,Delayed speech and language development,Cerebellar hypoplasia,Constipation,Developmental regression,Inappropriate crying |
| 00080821 | ME | m | 1.7 | Yes | Yes | PLA2G6 | NM_003560.2 | Infantile neuroaxonal dystrophy 1 (OMIM:256600) | AR | hom | Frameshift | c.1547_1548dup | p.(G517Rfs*29) | Inherited from parents | P | Irritability,Muscular hypotonia,Developmental regression,Abnormality of the cerebral white matter,Abnormality of the periventricular white matter,Brain atrophy |
| 00080822 | ME | m | 1.8 | Yes | No | WWOX | NM_016373.2 | Epileptic encephalopathy, early infantile, 28(OMIM:616211) | AR | hom | Splicing | c.409+1G>T | p.? | Inherited from parents | LP | Microcephaly,Low anterior hairline,Long philtrum,Low-set ears,Long eyelashes,Nystagmus,Synophrys,Seizures,Spasticity,Global developmental delay,Encephalopathy,Failure to thrive,Abnormal facial shape,Hepatomegaly,Kyphosis,Hyperbilirubinemia,Platyspondyly (childhood),Depressed nasal bridge,Hyperactive deep tendon reflexes,Mucopolysacchariduria,Central hypotonia,Brain atrophy,Cephalohematoma,Cerebral palsy |
| 00080823 | SA | m | unknown | No | Yes | USH1C | NM_153676.3 | Deafness, autosomal recessive 18A(OMIM:602092) | AR | hom | Nonsense | c.586C>T | p.(R196*) | Inherited from parents | LP | Hearing impairment |
| 00080824 | ME | m | 2.5 | Yes | No | KCTD3 | NM_016121.3 | Severe psychomotor retardation, seizures and cerebellar hypoplasia(PMID:25558065) | AR | hom | Frameshift | c.1036_1073del | p.(P346Tfs*4) | Inherited from parents | LP | Hydrocephalus,Delayed speech and language development,Seizures,Global developmental delay,Dandy-Walker malformation,Polymicrogyria,Abnormality of the cerebral white matter,Abnormal cortical gyration |
| 00080825 | ME | m | 3.9 | Yes | Yes | C12orf57 | NM_138425.2 | Temtamy syndrome(OMIM:218340) | AR | hom | Nonsense | c.1A>G | p.(M1?) | Inherited from parents | P | High palate,Abnormality of the face,Deeply set eye,Muscular hypotonia,Motor delay,Muscle weakness,Decreased fetal movement,Thrombocytosis,Polycythemia,Respiratory distress,Hypercalcemia,Lactic acidosis,Abnormality of mouth shape,Abnormality of brain morphology,Abnormality of muscle size |
| 00080826 | ME | m | 4.8 | No | N.i | SLC1A4 | NM_003038.4 | Spastic tetraplegia, thin corpus callosum, and progressive microcephaly (OMIM:616657) | AR | hom | Nonsense | c.573T>G | p.(Y191*) | N.i | LP | Microcephaly,Spasticity,Global developmental delay,Agenesis of corpus callosum,Failure to thrive,Constipation,Scoliosis,Brain atrophy |
| 00080827 | Am | f | 41.1 | N.i | Yes | LRSAM1 | NM_138361.5 | Charcot-Marie-Tooth disease, axonal, type 2P(OMIM:614436) | AD | het | Frameshift | c.2075_2087del | p.(H692Pfs*39) | Inherited from mother* | LP | Hypertension,Muscle weakness,Motor axonal neuropathy,Peripheral neuropathy,Abnormal levels of creatine kinase in blood |
| 00080828 | ME | m | 2.2 | N.i | N.i | CREBBP | NM_004380.2 | Rubinstein-Taybi syndrome(OMIM:180849) | AD | het | Splicing | c.2158+1G>A | p.? | N.i | LP | Global developmental delay,Growth delay,Abnormal facial shape,Bilateral cryptorchidism |
| 00080829 | ME | f | 7.2 | No | No | PEX6 | NM_000287.3 | Peroxisomebiogenesis disorder 4A(OMIM:614862) | AR | hom | Missense | c.1802G>A | p.(R601Q) | Inherited from parents | P | Spastic paraplegia |
| 00080830 | ME | f | 3.1 | Yes | No | MYO7A | NM_000260.3 | Usher syndrome, type 1B(OMIM:276900) | AR | hom | In-frame | c.5886_5888del | p.(F1963del) | Inherited from parents | P | Hearing impairment |
| 00080831 | ME | m | 0.6 | No | N.i | EPG5 | NM_020964.2 | Vici syndrome(OMIM:242840) | AR | hom | Nonsense | c.984dup | p.(K329*) | Inherited from parents | LP | Cataract,Agenesis of corpus callosum,Muscle weakness,Diarrhea,Heterotopia,Abnormal levels of creatine kinase in blood |
| 00080832 | ME | m | 5.3 | No | N.i | NF1 | NM_001042492.2 | Neurofibromatosis type 1(OMIM:162200) | AD | het | Missense | c.107C>G | p.(T36S) | Inherited from mother* | P | Tall stature,Autism,Muscular hypotonia |
| 00080833 | ME | f | 17.2 | N.i | No | CLDN16 | NM_006580.3 | Hypomagnesemia 3, renal type(OMIM:248250) | AR | hom | Nonsense | c.114C>A | p.(C38*) | N.i | LP | Psychosis,Seizures,Muscle weakness,Respiratory failure,Reduced dihydropyrimidine dehydrogenase activity,Decreased body weight,Reduced consciousness/confusion,Lower limb muscle weakness |
| 00080834 | ME | m | 3.1 | Yes | N.i | PKHD1 | NM_138694.3 | Polycystic kidney and hepatic disease(OMIM:263200) | AR | hom | Missense | c.4870C>T | p.(R1624W) | Inherited from parents | P | Enlarged kidneys,Macrocephaly,Delayed speech and language development,Motor delay,Frontal bossing,Constipation,Hypoglycemic seizures,Hepatomegaly |
| 00080835 | ME | m | 1.0 | No | N.i | PGK1 | NM_000291.3 | Phosphoglycerate kinase 1 deficiency(OMIM:300653) | X-linked | hemi | Missense | c.758T>C | p.(I253T) | inherited from mother | P | Abnormality of the face,Triangular face,Long philtrum,Thick eyebrow,Long palpebral fissure,Congenital diaphragmatic hernia,Spasticity,Motor delay,Patent ductus arteriosus,Abnormality of the lung,Abnormality of the vertebrae,Abnormality of movement,Hernia |
| 00080836 | SA | m | 2.7 | No | N.i | MFN2 | NM_014874.3 | Charcot-Marie-Tooth disease, type 2A2(OMIM:609260) | AD | het | Missense | c.250A>G | p.(K84E) | De Novo | P | Abnormality of eye movement,Nystagmus,Split hand,Seizures,Muscular hypotonia,Spasticity,Global developmental delay,Areflexia,Flexion contracture,Pes cavus,Distal amyotrophy,Motor polyneuropathy,Progressive pes cavus |
| 00080837 | ME | m | 1.0 | Yes | No | KCNQ2 | NM_172107.2 | Epileptic encephalopathy, early infantile, 7(OMIM:613720) | AD | het | Missense | c.1744A>T | p.(I582F) | De novo | LP | Seizures,Motor delay,Epileptic encephalopathy |
| 00080838 | ME | m | 1.1 | No | Yes | CDK5RAP2 | NM_018249.5 | Microcephaly 3, primary, autosomal recessive(OMIM:604804) | AR | hom | Nonsense | c.1865C>G | p.(S622*) | Inherited from parents | LP | Microcephaly,Abnormality of the face,Rod-cone dystrophy,Nystagmus,Global developmental delay |
| 00080839 | SA | f | 7.6 | No | Yes | CRLF1 | NM_004750.4 | Cold-induced sweating syndrome 1(OMIM:272430) | AR | hom | Splicing | c.397+1G>A | p.? | Inherited from parents | P | Seizures,Motor delay,Muscle weakness,Oligohydramnios,Myopathic facies,Recurrent infections,Myopathy,Aplasia/Hypoplasia of the nails,Neonatal asphyxia |
| 00080840 | ME | m | 9.4 | Yes | Yes | OPA1 | NM_130837.2 | Optic atrophy 1(OMIM:165500) | AD | het | Missense | c.1499G>A | p.(R500H) | De Novo | P | Optic atrophy,Motor delay |
| 00080841 | ME | m | 2.9 | Yes | Yes | F7 | NM_000131.4 | Factor VII deficiency(OMIM:227500) | AR | hom | Missense | c.920G>A | p.(R307H) | Inherited from parents | P | Microcephaly,Blepharospasm,Spasticity,Global developmental delay,Abnormality of coagulation,Generalized seizures |
| 00080842 | ME | u | prenatal | Yes | Yes | RYR1 | NM_000540.2 | Minicore myopathy with external ophthalmoplegia(OMIM:255320) | AR | hom | Frameshift | c.2476_2485del | p.(K826Dfs*55) | Inherited from parents | LP | Wrist flexion contracture,Abnormal joint morphology,Hydrops fetalis,Nonimmune hydrops fetalis,Fetal akinesia sequence,Hypokinesia,Multiple joint contractures,Limb undergrowth,Fetal cystic hygroma |
| 00080843 | ME | f | 2.0 | No | Yes | TECTA | NM_005422.2 | Deafness, autosomal recessive 21 (OMIM:603629) | AR | hom | Splicing | c.4690-1G>A | p.? | Inherited from parents | LP | Epicanthus,Sensorineural hearing impairment,Wide nasal bridge,Anteverted nares,Blue sclerae |
| 00080844 | ME | u | unknown | No | No | IDS | NM_000202.5 | Mucopolysaccharidosis II(OMIM:309900) | X | hemi | Missense | c.934G>A | p.(G312S) | Inherited from mother | LP | Inguinal hernia,Cryptorchidism,Abnormality of the hand,Muscle weakness,Decreased fetal movement,Abnormality of the foot,Talipes equinovarus,Rigidity,Scoliosis,Arthrogryposis multiplex congenita,Hip dislocation,Multiple joint contractures,Short stature,Decreased body weight |
| 00080845 | ME | m | 0.7 | Yes | Yes | IGHMBP2 | NM_002180.2 | Neuronopathy, distal hereditary motor, type VI(OMIM:604320) | AR | hom | Nonsense | c.127C>T | p.(R43*) | Inherited from parents | P | Microcephaly,Seizures,Muscular hypotonia,Reduced tendon reflexes,Peripheral axonal neuropathy,Respiratory failure requiring assisted ventilation,Foot dorsiflexor weakness |
| 00080846 | ME | m | 7.5 | Yes | No | RELN | NM_005045.3 | Lissencephaly 2 (Norman-Roberts type)(OMIM:257320) | AR | hom | Frameshift | c.9841del | p.(A3281Qfs*11) | Inherited from parents | LP | Delayed speech and language development,Intellectual disability,Spasticity,Motor delay,Lissencephaly,Hyperreflexia,Tetralogy of Fallot,Generalized seizures |
| 00080847 | ME | f | 1.7 | Yes | No | ACADVL | NM_001270447.1 | VLCAD deficiency(OMIM:201475) | AR | hom | Missense | c.494T>C | p.(F165S) | Inherited from parents | LP | Microcephaly,Global developmental delay,Failure to thrive,Lactic acidosis,CNS demyelination,Brain atrophy |
| 00080848 | ME | m | 0.7 | Yes | N.i | CYP21A2 | NM_000500.7 | Adrenal hyperplasia, congenital, due to 21-hydroxylase deficiency(OMIM:201910) | AR | hom | Missense | c.850A>G | p.(M284V) | Inherited from parents | P | Abnormality of the face,Muscular hypotonia,Lethargy,Umbilical hernia,Metabolic acidosis,Vomiting,Diarrhea,Hernia |
| 00080849 | ME | f | 24.7 | Yes | Yes | C12orf57 | NM_138425.2 | Temtamy syndrome(OMIM:218340) | AR | hom | Nonsense | c.1A>G | p.(M1?) | Inherited from parents | P | Autism,Delayed speech and language development,Intellectual disability,Muscular hypotonia,Global developmental delay,Agenesis of corpus callosum |
| 00080850 | ME | m | 15.6 | No | N.i | RIT1 | NM_001256821.1 | Noonan syndrome 8(OMIM:615355) | AD | het | Missense | c.295T>G | p.(F99V) | De Novo | P | obsolete Malformation of the heart and great vessels,Thickened calvaria,Short stature |
| 00080851 | ME | u | unknown | Yes | Yes | GBA | NM_000157.3 | Gaucher disease, perinatal lethal(OMIM:608013) | AR | hom | Missense | c.820G>A | p.(E274K) | Inherited from parents | P | Edema,Talipes equinovarus,Pulmonary hypoplasia,Spontaneous abortion,Low maternal serum estriol |
| 00080852 | Am | m | 3.5 | N.i | N.i | MED12 | NM_005120.2 | Opitz-Kaveggia syndrome(OMIM:305450) | X | hemi | Missense | c.2881C>T | p.(R961W) | Inherited from mother | P | Cryptorchidism,Thin upper lip vermilion,Macrocephaly,Abnormality of the face,Hypertelorism,Low-set ears,Wide nasal bridge,Short neck,Seizures,Global developmental delay,Cardiomyopathy,obsolete Malformation of the heart and great vessels,Short stature,Abnormality of the palpebral fissures,Abnormality of the external nose,Pointed helix,Aplasia/Hypoplasia of the eyebrow |
| 00080853 | ME | m | 2.0 | Yes | No | DLD | NM_000108.3 | Dihydrolipoamide dehydrogenase deficiency(OMIM:246900) | AR | hom | Missense | c.1436A>T | p.(D479V) | Inherited from parents | P | Inguinal hernia,Hypospadias,Microcephaly,Abnormality of the optic nerve,Nystagmus,Optic atrophy,Seizures,Global developmental delay,Motor delay,Abnormality of the basal ganglia,Lactic acidosis,Generalized muscle weakness,Hyperalaninemia,Increased serum pyruvate,Focal seizures,Central hypotonia |
| 00080854 | SA | m | unknown | No | Yes | GIPC3 | NM_133261.2 | Deafness, autosomal recessive 15(OMIM:601869) | AR | hom | Splicing | c.412-1G>T | p.? | Inherited from parents | LP | Hearing impairment |
| 00080855 | ME | m | 3.1 | Yes | No | DSG4 | NM_001134453.1 | Hypotrichosis 6(OMIM:607903) | AR | hom | Nonsense | c.2446C>T | p.(R816*) | Inherited from parents | LP | Abnormality of the hair,Alopecia,Sparse scalp hair,Ichthyosis |
| 00080856 | ME | m | 4.6 | N.i | N.i | CHD7 | NM_017780.3 | CHARGE syndrome(OMIM:214800) | AD | het | Nonsense | c.2959C>T | p.(R987*) | De novo | P | Hearing impairment,Abnormality of the ear,Autism,Delayed speech and language development,Syndactyly,Intellectual disability,Motor delay,Agenesis of corpus callosum,Failure to thrive,Cardiomyopathy,Developmental regression,Postnatal growth retardation,Brain atrophy |
| 00080857 | Am | f | 16.4 | N.i | N.i | NRAS | NM_002524.4 | autoimmune lymphoproliferative syndrome type IV, somatic (OMIM:614470) | AD | het | Missense | c.35G>A | p.(G12D) | De novo | LP | Nephritis,Ovarian cyst,Sinusitis,Depression,Abnormality of the mitral valve,Diarrhea,Abdominal pain,Pneumonia,Recurrent infections,Immunodeficiency,Recurrent abscess formation,Systemic lupus erythematosus,Myopathy,Recurrent bacterial skin infections,Abnormality of the fallopian tube,Hemorrhagic ovarian cyst,Clinodactyly,Endocarditis |
| 00080858 | ME | m | 9.0 | Yes | No | FBP1 | NM_000067.2 | Fructose-1,6-bisphosphatase deficiency(OMIM:229700) | AR | hom | In-frame | c.114_119dup | p.(C39_T40dup) | Inherited from mother& | P | Muscular hypotonia,Motor delay,Hepatic steatosis,Metabolic acidosis,Hypoglycemia,Hepatomegaly,Brain atrophy |
| 00080859 | ME | f | 3.9 | Yes | No | COL6A3 | NM_001197104.1 | Ullrich congenital muscular dystrophy 1(OMIM:254090) | AR | hom | Frameshift | c.8136del | p.(R2713Gfs*3) | Inherited from parents | LP | Muscular hypotonia,Motor delay,Muscle weakness |
| 00080860 | ME | m | 12.2 | Yes | Yes | SCN9A | NM_002977.3 | HSAN2D, autosomal recessive (OMIM:243000) | AR | hom | Nonsense | c.3503G>A | p.(W1168*) | Inherited from parents | LP | Eczema,Pallor,Atypical scarring of skin,Osteomyelitis,Short stature,Abnormal blistering of the skin,Peripheral neuropathy,Abnormality of pain sensation |
| 00080861 | ME | u | unknown | No | Yes | CSPP1 | NM_024790.6 | Joubert syndrome 21 (OMIM:615636) | AR | hom | Frameshift | c.2244_2247del | p.(E750Kfs*7) | Inherited from parents | P | Abnormality of upper lip,Encephalocele |
| 00080862 | Eu | f | 4.3 | No | No | SCN1A | NM_001165963.1 | Dravet syndrome(OMIM:607208) | AD | het | Missense | c.1261G>A | p.(V421M) | De Novo | LP | Neurodegeneration |
| 00080863 | SA | f | 8.1 | N.i | N.i | POLG | NM_002693.2 | Progressive external ophthalmoplegia, autosomal recessive 1 (OMIM:258450) | AR | hom | Missense | c.911T>G | p.(L304R) | Inherited from parents | P | Ptosis,Progressive external ophthalmoplegia,Increased serum lactate,Ragged-red muscle fibers,Elevated serum creatine phosphokinase,Decreased activity of cytochrome C oxidase in muscle tissue |
| 00080864 | Eu | m | 5.5 | No | Yes | TGFB3 | NM_003239.2 | Loeys-Dietz syndrome type 5(OMIM:615582) | AD | het | Missense | c.898C>T | p.(R300W) | Inherited from father* | P | Inguinal hernia,Cleft palate,Muscular hypotonia,Joint hypermobility,Pes planus |
| 00080865 | ME | m | 9.7 | No | N.i | RBBP8 | NM_001039885.2 | Seckel syndrome type 2(OMIM:606744) | AR | hom | Missense | c.298C>T | p.(R100W) | N.i | P | Micropenis,Delayed speech and language development,Hyperactivity,Global developmental delay,Obesity,Insomnia |
| 00080866 | ME | f | 0.8 | No | N.i | FBXL4 | NM_001278716.1 | m DNA depletion syndrome 13 (encephalomyopathic type)(OMIM:615471) | AR | hom | Frameshift | c.1648_1649del | p.(D550Hfs*2) | Inherited from parents | LP | Abnormality of Krebs cycle metabolism,Lactic acidosis |
| 00080867 | ME | m | 4.6 | Yes | Yes | ASNS | NM_183356.3 | Asparagine synthetase deficiency(OMIM:615574) | AR | hom | Missense | c.1193A>G | p.(Y398C) | Inherited from parents | P | Microcephaly,Abnormality of the face,Micrognathia,Seizures,Global developmental delay,Vomiting,Progressive spastic quadriplegia,Spastic tetraplegia |
| 00080868 | ME | u | prenatal | No | N.i | SLC26A2 | NM_000112.3 | Achondrogenesis Ib(OMIM:600972) | AR | hom | Missense | c.1448T>C | p.(L483P) | Inherited from parents | P | Aplasia/hypoplasia of the extremities |
| 00080869 | ME | f | 0.6 | Yes | No | TTN | NM_000282.3 | Myopathy, early-onset, with fatal cardiomyopathy(OMIM:611705) | AR | hom | Splicing | c.24730+1G>T | p.? | Inherited from parents | LP | Abnormality of the face,Full cheeks,Sloping forehead,Micrognathia,Small forehead,Narrow nose,Short neck,Thickened nuchal skin fold,Muscular hypotonia,Global developmental delay,Hyporeflexia,Failure to thrive,Leukodystrophy,Abnormality of serum amino acid levels,Limb joint contracture,Lactic acidosis,Depressed nasal bridge,Tapering pointed ends of distal finger phalanges,Abnormal hair pattern,Abnormality of serine family amino acid metabolism,Brain atrophy,Abnormal myelination,Heart murmur |
| 00080870 | ME | m | 15.8 | Yes | Yes | TTN | NM_001178008.1 | Myopathy, early-onset, with fatal cardiomyopathy(OMIM:611705) | AR | hom | Splicing | c.98671-2A>G | p.? | Inherited from parents | LP | Muscle weakness,Abnormal levels of creatine kinase in blood |
| 00080871 | ME | m | 5.2 | No | N.i | CA2 | NM_000067.2 | Osteopetrosis, autosomal recessive 3, with renal tubular acidosis(OMIM:259730) | AR | hom | Splicing | c.232+1G>A | p.? | N.i | P | Abnormality of eye movement,Muscular hypotonia,Global developmental delay |
| 00080872 | ME | m | 0.9 | Yes | N.i | WDR81 | NM_001163809.1 | Cerebellar ataxia, mental retardation, and dysequilibrium syndrome 2(OMIM:610185) | AR | hom | Frameshift | c.850_851del | p.(L284Vfs*9) | Inherited from parents | LP | Hydrocephalus,Abnormality of the face,Cataract,Holoprosencephaly,Atria septal defect,Splenomegaly,Hepatomegaly,Abnormality of brain morphology |
| 00080873 | ME | f | 7.2 | No | No | KMT2A | NM_001197104.1 | Wiedemann-Steiner syndrome(OMIM:605130) | AD | het | Missense | c.3473G>A | p.(C1158T) | De Novo | LP | Abnormality of the face,Global developmental delay,Intellectual disability, severe |
| 00080874 | ME | f | 1.2 | No | N.i | IGHMBP2 | NM_002180.2 | Charcot-Marie-Tooth disease, axonal, type 2S (OMIM:616155) | AR | hom | Nonsense | c.958C>T | p.(R320*) | Inherited from parents | P | Motor delay,Developmental regression,Brain atrophy |
| 00080875 | Eu | f | 42.2 | No | N.i | SLC12A3 | NM_000339.2 | Gitelman syndrome(OMIM:263800) | AR | hom | Missense | c.1315G>A | p.(G439S) | Inherited from parents | P | Abnormality of metabolism/homeostasis,Short stature,Abnormal renal physiology |
| 00080876 | ME | f | 6.2 | No | No | C12orf57 | NM_138425.2 | Temtamy syndrome(OMIM:218340) | AR | hom | Nonsense | c.1A>G | p.(M1?) | Inherited from parents | P | Abnormality of the face,Nystagmus,Autism,Ataxia,Muscular hypotonia,Global developmental delay,Gait disturbance |
| 00080877 | ME | f | 7.8 | Yes | No | GALT | NM_000155.3 | Galactosemia(OMIM:230400) | AR | hom | Missense | c.404C>T | p.(S135L) | Inherited from parents | P | Cataract,Cyanosis,Global developmental delay,Failure to thrive,Dysphagia,Apnea |
| 00080878 | ME | u | prenatal | No | N.i | CEP290 | NM_025114.3 | Joubert syndrome 5(OMIM:610188) | AR | hom | Nonsense | c.3894dup | p.(K1299*) | Inherited from parents | LP | Abnormality of the kidney |
| 00080879 | ME | f | 1.9 | Yes | Yes | CYP2U1 | NM_183075.2 | Spastic paraplegia 56, autosomal recessive(OMIM:615030) | AR | hom | Missense | c.947A>T | p.(D316V) | Inherited from parents | P | Cafe-au-lait spot,Abnormality of skin pigmentation,Spasticity,Motor delay,Clonus,Abnormality of the cerebral white matter |
| 00080880 | ME | m | 3.8 | Yes | Yes | SLC2A10 | NM_030777.3 | Arterial tortuosity syndrome(OMIM:208050) | AR | hom | Missense | c.243C>G | p.(S81R) | Inherited from parents | P | Inguinal hernia,Congenital diaphragmatic hernia,Joint hypermobility,Joint laxity,Aortic dilatation,Arterial tortuosity,Abnormally lax or hyperextensible skin,Hernia |
| 00080881 | ME | m | 8.2 | Yes | Yes | C12orf57 | NM_138425.2 | Temtamy syndrome(OMIM:218340) | AR | hom | Nonsense | c.1A>G | p.(M1?) | Inherited from parents | P | Hydrocephalus,Macrocephaly,Coloboma,Optic atrophy,Hypothyroidism,Agenesis of corpus callosum,Muscle weakness,Constipation |
| 00080882 | Eu | f | 0.7 | No | Yes | MEFV | NM_000243.2 | Familial Mediterranean fever, AR(OMIM:249100) | AR | hom | Missense | c.2080A>G | p.(M694V) | Inherited from parents | P | Nystagmus,Muscular hypotonia,Agenesis of corpus callosum,Splenomegaly,Hepatomegaly,Brain atrophy,Abnormal levels of creatine kinase in blood |
| 00080883 | Eu | m | 3.4 | No | N.i | ANKRD11 | NM_001256182.1 | KBG syndrome(OMIM:148050) | AD | het | Splicing | c.7570-2A>G | p.? | De Novo | LP | Global developmental delay,Duane anomaly |
| 00080884 | ME | m | 3.1 | Yes | N.i | ADAT3 | NM_138422.2 | Mental retardation, autosomal recessive 36(OMIM:615286) | AR | hom | Missense | c.430G>A | p.(V144M) | Inherited from parents | P | Abnormality of the face,Global developmental delay |
| 00080885 | Eu | u | unknown | No | Yes | GUSB | NM_000181.3 | Mucopolysaccharidosis VII(OMIM:253220) | AR | hom | Missense | c.1484A>G | p.(Y495C) | Inherited from parents | P | Hydrops fetalis,Short nose,Abnormality of the columella,Abnormality of limbs |
| 00080886 | ME | m | 1.3 | Yes | Yes | SLC52A3 | NM_033409.3 | Fazio-Londe disease(OMIM:211500) | AR | hom | Nonsense | c.71G>A | p.(W24*) | Inherited from parents | LP | Ptosis,Global developmental delay,Myocardial infarction,Abnormality of serum amino acid levels,Feeding difficulties,Abnormal basal ganglia MRI signal intensity |
| 00080887 | ME | m | 4.5 | No | N.i | CLCNKB | NM_000085.4 | Bartter syndrome, type 4B(OMIM:607364) | AR | hom | Large Del | c.-152_*410del | p.? | Inherited from parents | P | Global developmental delay,Failure to thrive,Episodic vomiting |
| 00080888 | ME | f | 1.1 | Yes | Yes | SNAP29 | NM_004782.3 | Cerebral dysgenesis, neuropathy, ichthyosis, and palmoplantar keratoderma syndrome(OMIM:609528) | AR | hom | Frameshift | c.223del | p.(V75Sfs*28) | Inherited from parents | P | Nystagmus,Intellectual disability,Muscle weakness,Ichthyosis,Postnatal growth retardation,Cortical visual impairment |
| 00080889 | ME | m | 1.3 | No | No | SLC2A1 | NM_006516.2 | GLUT1 deficiency syndrome 1(OMIM:606777) | AD | het | Splicing | c.1279-2A>C | p.? | De Novo | LP | Microcephaly,Intellectual disability,Global developmental delay,Generalized seizures,Short stature,Neurodevelopmental delay |
| 00080890 | ME | u | unknown | N.i | Yes | POMGNT2 | NM_032806.5 | Muscular dystrophy-dystroglycanopathy (congenital with brain and eye anomalies, type A, 8(OMIM:614830) | AR | hom | Missense | c.473G>A | p.(R158H) | Inherited from parents | LP | Bilateral microphthalmos,Abnormal brainstem MRI signal intensity |
| 00080891 | ME | f | 0.8 | Yes | Yes | HSD3B7 | NM_025193.3 | Bile acid synthesis defect, congenital, 1 (OMIM:607765) | AR | hom | Splicing | c.694+2del | p.? | Inherited from parents | LP | Jaundice,Cholestasis |
| 00080892 | Eu | m | 4.4 | No | No | MAGEL2 | NM_019066.4 | Schaaf-Yang syndrome(OMIM:615547) | AD | het | Frameshift | c.1996dup | p.(Q666Pfs*47) | De novo | P | Cryptorchidism,Scrotal hypoplasia,Micropenis,Microcephaly,Low-set ears,Nystagmus,Cafe-au-lait spot,Muscular hypotonia,Flexion contracture,Talipes,Abnormal facial shape,Recurrent lower respiratory tract infections,Reduced visual acuity,Sparse hair,Severe global developmental delay,Feeding difficulties |
| 00080893 | ME | m | 6.3 | Yes | Yes | COL6A2 | NM_001849.3 | Ullrich congenital muscular dystrophy 1(OMIM:254090) | AR | hom | Splicing | c.2461+1G>A | p.? | Inherited from parents | LP | Osteopenia,Intellectual disability,Muscular hypotonia,Global developmental delay,Joint hypermobility,Abnormal facial shape,Short stature,Finger joint hypermobility,Hypermobility of toe joints |
| 00080894 | Am | m | 22.3 | No | Yes | CLCN1 | NM_000083.2 | Myotonia congenita, recessive(OMIM:255700) | AR | hom | Nonsense | c.1129C>T | p.(R377*) | Inherited from parents | P | Muscle weakness,Dystonia,Rigidity,Decreased activity of cytochrome C oxidase in muscle tissue,Proximal muscle weakness,Congenital muscular dystrophy,Abnormality of muscle fibers |
| 00080895 | ME | m | 5.2 | Yes | Yes | PSAP | NM_001042465.1 | Combined SAP deficiency(OMIM:611721) | AR | hom | Missense | c.722G>C | p.(C241S) | Inherited from parents | P | Hydronephrosis,Macrocephaly,Triangular face,Muscular hypotonia,Abnormal facial shape,Unsteady gait,Developmental regression,Diffuse white matter abnormalities,Infantile axial hypotonia,Abnormality of muscle size |
| 00080896 | ME | f | 4.7 | No | No | PDHA1 | NM_001173454.1 | Pyruvate dehydrogenase E1-alpha deficiency(OMIM:312170) | X-linked | het | Frameshift | c.1256_1259dup | p.(W421Sfs*6) | De Novo | P | Muscular hypotonia,Global developmental delay,Leukodystrophy,Abnormality of the cerebral white matter,Lactic acidosis,Abnormal myelination |
| 00080897 | ME | m | 2.1 | Yes | No | LARP7 | NM_015454.2 | Alazami syndrome(OMIM:615071) | AR | hom | Frameshift | c.1024_1030dup | p.(T344Kfs*9) | Inherited from parents | P | Cleft palate,Microcephaly,Delayed speech and language development,Global developmental delay,Failure to thrive,Intrauterine growth retardation |
| 00080898 | Eu | f | 1.3 | No | N.i | PURA | NM_005859.4 | Mental retardation, autosomal dominant 31(OMIM:616158) | AD | het | Frameshift | c.502del | p.(L168Cfs*57) | De Novo | LP | Abnormality of the kidney,Microcephaly,Abnormality of the endocrine system,Intellectual disability,Seizures,Muscle weakness,Leukodystrophy |
| 00080899 | ME | m | 12.7 | Yes | Yes | MPLKIP | NM_138701.3 | Trichothiodystrophy 4, nonphotosensitive (OMIM:234050) | AR | hom | Nonsense | c.85G>T | p.(G29*) | Inherited from parents | LP | High palate,Hyperkeratosis,Prominent fingertip pads,Intellectual disability,Spasticity,Hypoplasia of the frontal lobes,Bilateral single transverse palmar creases,Prominent epicanthal folds |
| 00080900 | ME | f | 4.4 | Yes | Yes | OCLN | NM_002538.3 | Band-like calcification with simplified gyration and polymicrogyria (OMIM:251290) | AR | hom | Frameshift | c.173_194del | p.(W58Ffs*10) | Inherited from parents | LP | Microcephaly,Delayed speech and language development,Intellectual disability,Motor delay,Muscle weakness,Growth delay,Abnormal facial shape,Generalized seizures,Developmental regression,Elevated hepatic transaminases,Lactic acidosis,Epileptic spasms,Neurodevelopmental delay |
| 00080901 | ME | f | 3.0 | N.i | No | MECP2 | NM_001110792.1 | Rett syndrome(OMIM:312750) | X | het | Nonsense | c.916C>T | p.(R306*) | De Novo | P | Delayed speech and language development,Global developmental delay,Generalized hypotonia |
| 00080902 | ME | m | 1.4 | Yes | No | ALDH7A1 | NM_001182.4 | Epilepsy, pyridoxine-dependent(OMIM:266100) | AR | hom | Nonsense | c.328C>T | p.(R110*) | Inherited from parents | P | Nystagmus,Irritability,Seizures,Muscular hypotonia,Global developmental delay,Motor delay,Cerebellar vermis hypoplasia,Hyperreflexia,Metabolic acidosis,Ventriculomegaly,Status epilepticus,EEG abnormality,Abnormality of the cerebral white matter,Abnormality of the periventricular white matter,Disseminated intravascular coagulation,Periventricular leukomalacia,Fetal choroid plexus cysts,Elevated urinary homovanillic acid,Hypoxemia,Abnormal brainstem MRI signal intensity,Abnormality of movement,Sepsis |
| 00080903 | Eu | m | 2.4 | No | No | ARID1B | NM_020732.3 | Mental retardation, autosomal dominant 12(OMIM:614562) | AD | het | Frameshift | c.5547dup | p.(S1851Kfs*5) | De Novo | LP | Global developmental delay,Delayed speech and language development,Abnormal facial shape,Retractile testis,Apnea,Bradycardia,febrile seizures |
| 00080904 | ME | f | 3.7 | No | N.i | MYO7A | NM_000260.3 | Usher syndrome, type 1B(OMIM:276900) | AR | hom | Missense | c.5617C>T | p.(R1873W) | Inherited from parents | P | Hearing impairment |
| 00080905 | ME | f | 1.7 | No | Yes | DYNC1H1 | NM_001376.4 | Mental retardation, autosomal dominant 13(OMIM:614563) | AD | het | Missense | c.9250G>A | p.(A3084T) | De Novo | LP | Microcephaly,Abnormality of the face,Sloping forehead,Abnormality of the pinna,Delayed speech and language development,Large eyes,Global developmental delay,Motor delay,Alkalosis,Highly arched eyebrow,Hip dislocation,External ear malformation,Polydactyly,Feeding difficulties,Methylmalonic aciduria |
| 00080906 | ME | m | 37.3 | No | Yes | COLQ | NM_005677.3 | Myasthenic syndrome, congenital, 5(OMIM:603034) | AR | hom | Missense | c.1250G>A | p.(C417Y) | N.i | P | Myopathy |
| 00080907 | ME | m | 1.0 | No | No | KMT2A | NM_001197104.1 | Wiedemann-Steiner syndrome (OMIM:605130) | AD | het | Nonsense | c.5251A>T | p.(K1751*) | De Novo | LP | Microcephaly,Abnormality of the pharynx,Delayed speech and language development,Muscular hypotonia,Global developmental delay,Motor delay,Failure to thrive,Growth delay,Bicuspid aortic valve,Abnormal facial shape,Feeding difficulties |
| 00080908 | ME | f | 2.4 | No | N.i | RECQL4 | NM_004260.3 | Rothmund-Thomson syndrome(OMIM:268400) | AR | hom | Nonsense | c.1000G>T | p.(E334*) | N.i | LP | Macrocephaly,Short stature |
| 00080909 | ME | m | 0.3 | No | Yes | NPC2 | NM_006432.3 | Niemann-pick disease, type C2(OMIM:607625) | AR | hom | Missense | c.278G>T | p.(C93F) | Inherited from parents | P | Hepatic failure,Hepatosplenomegaly,Elevated hepatic transaminases |
| 00080910 | ME | f | 4.1 | No | Yes | P3H1 | NM_001243246.1 | Osteogenesis imperfecta, type VIII(OMIM:610915) | AR | hom | Splicing | c.*59G>A | p.? | N.i | P | Abnormality of the skeletal system |
| 00080911 | ME | m | 4.2 | No | Yes | COL4A5 | NM_033380.2 | Alport syndrome(OMIM:301050) | X-linked | hemi | Frameshift | c.4341del | p.(G1448Vfs*106) | Inherited from mother | LP | Abnormality of the kidney |
| 00080912 | ME | m | 1.6 | Yes | Yes | FANCG | NM_004629.1 | Fanconi anemia, complementation group G(OMIM:614082) | AR | hom | Frameshift | c.637_643del | p.(Y213Kfs*6) | Inherited from parents | P | Abnormality of the kidney,Cholestasis,Hepatic failure,Hepatosplenomegaly,Anemia,Elevated hepatic transaminases |
| 00080913 | ME | f | 17.3 | No | N.i | SHOC2 | NM_007373.3 | Noonan-like syndrome with loose anagen hair(OMIM:607721) | AD | het | Missense | c.4A>G | p.(S2G) | De Novo | P | Global developmental delay,Ventricular septal defect |
| 00080914 | ME | f | 2.0 | Yes | N.i | FBXL4 | NM_012160.4 | m DNA depletion syndrome 13 (encephalomyopathic type)(OMIM:615471) | AR | hom | Nonsense | c.616C>T | p.(R206*) | Inherited from parents | LP | Delayed speech and language development,Muscular hypotonia,Global developmental delay,Motor delay,Muscle weakness,Failure to thrive,Increased CSF lactate,Lactic acidosis,Hyperalaninemia,Increased serum pyruvate |
| 00080915 | Am | m | 12.8 | No | No | SATB2 | NM_015265.3 | Glass syndrome(OMIM:612313) | AD | het | Splicing | c.1542+1G>A | p.? | De Novo | LP | High palate,Abnormality of the face,Autism,Delayed speech and language development,Seizures,Motor delay,Gait disturbance,Muscle weakness,Abnormality of the cerebral white matter,Depressed nasal bridge,Speech apraxia |
| 00080916 | ME | f | 3.9 | Yes | N.i | C12orf65 | NM_152269.4 | Combined oxidative phosphorylation deficiency 7(OMIM:613559) | AR | hom | Frameshift | c.248del | p.(V83Gfs*2) | Inherited from parents | P | Microcephaly,Nystagmus,Global developmental delay,Muscle weakness,Cardiomyopathy,Constipation,Lactic acidosis,Colpocephaly |
| 00080917 | ME | m | 2.8 | No | No | CTNNB1 | NM_001098210.1 | Mental retardation, autosomal dominant 19 (OMIM:615075) | AD | het | Frameshift | c.1925_1926del | p.(E642Vfs*5) | De Novo | LP | Microcephaly,Strabismus,Ophthalmoplegia,Delayed speech and language development,Intellectual disability,Spasticity,Global developmental delay,Motor delay,Hypertonia,Failure to thrive,Anemia,Abnormal facial shape,Short stature,Abnormal pyramidal signs,Postnatal growth retardation,Abnormality of movement |
| 00080918 | ME | m | 6.1 | No | Yes | CA2 | NM_000067.2 | Osteopetrosis, autosomal recessive 3, with renal tubular acidosis(OMIM:259730) | AR | hom | Nonsense | c.670A>T | p.(K224*) | Inherited from parents | LP | Abnormality of the face,Failure to thrive,Dysphagia,Dysharmonic delayed bone age,Abnormal appendicular skeleton morphology |
| 00080919 | SA | f | 1.0 | No | Yes | RMND1 | NM_017909.3 | Combined oxidative phosphorylation deficiency 11(OMIM:614922) | AR | hom | stoploss | c.1349G>C | p.(*450Sext*31) | Inherited from parents | P | Abnormality of the kidney,Renal tubular dysfunction,Global developmental delay,Motor delay,Muscle weakness,Failure to thrive,Metabolic acidosis,Hyperkalemia,Increased CSF lactate,Lactic acidosis,Abnormality of the mitochondrion |
| 00080920 | ME | m | 3.1 | Yes | Yes | PSAP | NM_001042465.1 | Combined SAP deficiency(OMIM:611721) | AR | hom | Missense | c.722G>C | p.(C241S) | Inherited from parents | P | Strabismus,Unsteady gait,Abnormality of the periventricular white matter |
| 00080921 | ME | u | unknown | No | No | COL1A2 | NM_000089.3 | Osteogenesis imperfecta, type III (OMIM:259420) | AD | het | Missense | c.2260G>T | p.(G754C) | De Novo | P | Abnormality of the thorax,Redundant skin,Breech presentation,Overlapping toe,Tibial bowing,Decreased skull ossification,Multiple prenatal fractures,Asymmetry of the shape of the ears |
| 00080922 | Eu | f | 4.0 | Yes | Yes | PCK1 | NM_002591.3 | Phosphoenolpyruvate carboxykinase-1, cytosolic, deficiency(OMIM:261680) | AR | hom | Missense | c.925G>A | p.(G309R) | Inherited from parents | LP | Hypoglycemia,Organic aciduria,Elevated hepatic transaminases,Abnormal CSF lactate level |
| 00080923 | SA | f | 12.0 | Yes | Yes | CAPN3 | NM_000070.2 | Muscular dystrophy, limb-girdle, type 2A(OMIM:253600) | AR | hom | Nonsense | c.1313G>A | p.(W438*) | Inherited from parents | LP | Muscular hypotonia,Gowers sign,Scapular winging,Proximal muscle weakness,Limb-girdle muscular dystrophy |
| 00080924 | ME | f | 3.0 | No | N.i | CHD2 | NM_001271.3 | Epileptic encephalopathy, childhood-onset(OMIM:615369) | AD | het | Missense | c.2068C>T | p.(H690Y) | De Novo | LP | Delayed speech and language development,Seizures,Global developmental delay,Motor delay |
| 00080925 | ME | f | 6.0 | No | No | SLC17A5 | NM_012434.4 | Salla disease(OMIM:604369) | AR | hom | Missense | c.116G>A | p.(R39H) | Inherited from parents | LP | Abnormality of the face,Global developmental delay,Leukodystrophy,Abnormality of glycolysis,Abnormal brainstem MRI signal intensity |
| 00080926 | ME | f | 0.4 | No | No | PAX6 | NM_001604.4 | Aniridia(OMIM:106210) | AD | het | Missense | c.196T>C | p.(C66R) | De Novo | P | Abnormality of eye movement,Visual loss,Nystagmus,Delayed speech and language development,Muscular hypotonia,Spasticity,Global developmental delay,Motor delay,Muscle stiffness |
| 00080927 | ME | f | 0.8 | Yes | No | RAG1 | NM_000448.2 | Omenn syndrome(OMIM:603554) | AR | hom | Missense | c.1331C>T | p.(A444V) | Inherited from parents | P | Abnormality of skin pigmentation,Immunodeficiency,Severe combined immunodeficiency,Ichthyosis |
| 00080928 | ME | u | unknown | Yes | Yes | DOK7 | NM_173660.4 | Fetal akinesia deformation sequence(OMIM:208150) | AR | hom | Stoploss | c.1513T>G | p.(*505Gext*182) | Inherited from parents | LP | Hydronephrosis,Downslanted palpebral fissures,Missing ribs,Muscular hypotonia,Pulmonary hypoplasia,Multiple joint contractures,Diaphragmatic eventration |
| 00080929 | ME | m | 1.7 | Yes | No | ISCA2 | NM_194279.3 | Multiple m dysfunctions syndrome type 4(OMIM:616370) | AR | hom | Missense | c.229G>A | p.(G77S) | Inherited from parents | P | Nystagmus,Seizures,Muscular hypotonia,Hyperreflexia,Delayed gross motor development,Leukodystrophy,Poor head control,Abnormality of the cerebral white matter,Recurrent infections,Recurrent lower respiratory tract infections,Delayed fine motor development,Central hypotonia,Abnormal myelination,Delayed myelination |
| 00080930 | ME | m | 5.6 | Yes | Yes | RSPH4A | NM_001010892.2 | Ciliary dyskinesia, primary, 11 (OMIM:612649) | AR | hom | Nonsense | c.1558C>T | p.(R520*) | Inherited from parents | LP | Asthma,Recurrent lower respiratory tract infections,Bronchiolitis obliterans |
| 00080931 | ME | f | 5.6 | Yes | Yes | IQCB1 | NM_001023570.2 | Senior-Loken syndrome type 5(OMIM:609254) | AR | hom | Frameshift | c.1540_1541dup | p.(T516Afs*7) | Inherited from parents | LP | Autism,Leber optic atrophy,Intellectual disability,Eye poking |
| 00080932 | ME | f | 8.1 | yes | yes | BBS4 | NM_033028.4 | Bardet-Biedl syndrome 4(OMIM:615982) | AR | hom | splicing | c.157-2A>G | p.? | inherited from parents | P | Microcephaly, Optic atrophy, Delayed speech and language development, Intellectual disability, Seizures, Spasticity, Spastic paraplegia, Motor delay, Hypertonia, Atria septal defect, Myelopathy, Enlarged cisterna magna, Leukodystrophy, Polydactyly, Encephalomalacia, Cerebral palsy |
| 00080933 | ME | m | 4.2 | yes | yes | KCNJ10 | NM_002241.4 | SESAME syndrome(OMIM:612780) | AR | hom | missense | c.170C>T | p.(T57I) | inherited from parents | P | Delayed speech and language development, Abnormality of skin pigmentation, Intellectual disability, Seizures, Muscular hypotonia, Motor delay, Infantile muscular hypotonia |
| 00080934 | ME | m | 2.4 | yes | no | HEXB | NM_000521.3 | Sandhoff disease, infantile, juvenile, and adult forms(OMIM:268800) | AR | hom | splicing | c.1169+3_1169+10del | p.? | inherited from parents | P | Dolichocephaly, Horizontal nystagmus, Seizures, Spasticity, Global developmental delay, Encephalopathy, Absent speech, Gastroesophageal reflux, Lower limb spasticity, Developmental regression, Upper limb spasticity, Nasogastric tube feeding in infancy, Delayed myelination |
| 00080935 | ME | f | 5 | yes | no | KCNQ1 | NM_000218.2 | Jervell and Lange-Nielsen syndrome(OMIM:220400) | AR | hom | stopgain | c.1552C>T | p.(R518*) | inherited from parents | P | Sensorineural hearing impairment, Delayed speech and language development, Seizures, Anemia, Generalized seizures, Arrhythmia |
| 00080936 | ME | f | 1.2 | Yes | Yes | ASL | NM_000048.3 | Argininosuccinic aciduria(OMIM:207900) | AR | hom | Nonsense | c.1060C>T | p.(Q345*) | Inherited from parents | P | Global developmental delay,Failure to thrive,Hyperammonemia,Abnormal facial shape,Frontal bossing,Diarrhea,Hepatomegaly,Short stature |
| 00080937 | SA | m | 5.5 | Yes | Yes | MFSD8 | NM_152778.2 | Ceroid lipofuscinosis, neuronal, 7 (OMIM:610951) | AR | hom | Nonsense | c.616C>T | p.(Q206*) | Inherited from parents | LP | Optic atrophy,Spasticity,Developmental regression,Leukodystrophy |
| 00080938 | SA | m | 1.8 | Yes | No | RARS | NM_002887.3 | Leukodystrophy, hypomyelinating, 9(OMIM:616140) | AR | hom | Missense | c.1A>C | p.(M1?) | Inherited from parents | P | Strabismus,Spasticity,Global developmental delay,Cerebellar vermis hypoplasia,Cerebellar hypoplasia,Generalized seizures,Leukodystrophy,Abnormal myelination |
| 00080939 | ME | m | 0.7 | No | N.i | CTSA | NM_000308.2 | Galactosialidosis(OMIM:256540) | AR | hom | Splicing | c.655-2A>G | p.? | N.i | LP | Abnormality of the face,Narrow chest,Ascites,Splenomegaly,Talipes equinovarus,Hydrops fetalis,Thrombocytopenia,Abnormal appendicular skeleton morphology,Brain atrophy,Abnormality of limbs |
| 00080940 | ME | m | 2.2 | No | N.i | FKRP | NM_001039885.2 | Muscular dystrophy-dystroglycanopathy (congenital with or without mental retardation), type B, 5(OMIM:606612) | AR | hom | Missense | c.898G>A | p.(V300M) | Inherited from parents | P | Microcephaly,Global developmental delay,Lissencephaly |
| 00080941 | ME | m | 1.9 | No | N.i | RAB3GAP1 | NM_001172435.1 | Warburg micro syndrome 1(OMIM:600118) | AR | hom | Nonsense | c.559C>T | p.(R187*) | Inherited from parents | LP | Cataract,Global developmental delay,Paraplegia |
| 00080942 | Am | m | 46.6 | No | Yes | ATP1A3 | NM_152296.4 | CAPOS syndrome(OMIM:601338) | AD | het | Missense | c.2452G>A | p.(E818K) | De Novo | P | Hearing impairment,Visual loss,Optic atrophy,Ataxia,Aganglionic megacolon,Encephalitis,Abnormal appendicular skeleton morphology,Vitamin B12 deficiency |
| 00080943 | ME | f | 3.1 | Yes | Yes | ASPM | NM_018136.4 | Microcephaly 5, primary, autosomal recessive(OMIM:608716) | AR | hom | Nonsense | c.8017C>T | p.(Q2673*) | Inherited from parents | P | Microcephaly,Delayed speech and language development |
| 00080944 | ME | m | 10.0 | Yes | N.i | FKRP | NM_001039885.2 | Muscular dystrophy-dystroglycanopathy (congenital with or without mental retardation), type B, 5(OMIM:606612) | AR | hom | Missense | c.1012G>T | p.(V338L) | Inherited from parents | P | Myopathy,Muscular dystrophy,Abnormal levels of creatine kinase in blood |
| 00080945 | ME | m | 0.7 | No | No | NSD1 | NM_022455.4 | Sotos syndrome 1(OMIM:117550) | AD | het | Nonsense | c.2323C>T | p.(Q775*) | De Novo | P | Abnormality of the face,Global developmental delay,Hypertonia,Hyperreflexia,Hypoglycemia |
| 00080946 | ME | m | 6.9 | Yes | Yes | SNX14 | NM_153816.3 | Spinocerebellar ataxia, autosomal recessive 20(OMIM:616354) | AR | hom | Nonsense | c.2722C>T | p.(Q908*) | Inherited from parents | LP | Abnormality of the face,Delayed speech and language development,Spasticity,Global developmental delay,Hyperreflexia,Abnormality of the cerebellar vermis,Brain atrophy |
| 00080947 | ME | m | 1.3 | Yes | Yes | ETHE1 | NM_014297.3 | Ethylmalonic encephalopathy(OMIM:602473) | AR | hom | Frameshift | c.592dup | p.(H198Pfs*23) | Inherited from parents | P | Microcephaly,Pallor,Muscular hypotonia,Global developmental delay,Hyperreflexia,Failure to thrive,Metabolic acidosis,Abnormality of the basal ganglia,Abnormality of midbrain morphology,Lactic acidosis,Decreased activity of m complex IV,Brain atrophy |
| 00080948 | ME | m | 10.9 | No | Yes | C12orf57 | NM_138425.2 | Temtamy syndrome(OMIM:218340) | AR | hom | Nonsense | c.1A>G | p.(M1?) | Inherited from parents | P | Seizures,Global developmental delay,Agenesis of corpus callosum,Failure to thrive,Dilation of lateral ventricles |
| 00080949 | ME | f | 2.9 | Yes | N.i | SERAC1 | NM_032861.3 | 3-methylglutaconic aciduria with deafness, encephalopathy, and Leigh-like syndrome(OMIM:614739) | AR | hom | Frameshift | c.438del | p.(T147Rfs*22) | Inherited from parents | P | Jaundice,Intellectual disability,Muscular hypotonia,Global developmental delay,Dystonia,Failure to thrive,Organic aciduria,Chorea,Abnormality of the basal ganglia,Short stature |
| 00080950 | ME | m | 1.7 | No | N.i | MTM1 | NM_000252.2 | Myotubular myopathy, X-linked(OMIM:310400) | X-linked | hemi | Splicing | c.1261-10A>G | p.? | De Novo | P | Hydrocephalus,Abnormality of the skeletal system,Muscular hypotonia,Encephalopathy,Abnormality of the lung,Intracranial hemorrhage |
| 00080951 | ME | f | 14.6 | Yes | Yes | PCNT | NM_006031.5 | Microcephalic osteodysplastic primordial dwarfism, type II(OMIM:210720) | AR | hom | Nonsense | c.658G>T | p.(E220*) | Inherited from parents | P | Microcephaly,Short stature |
| 00080952 | ME | m | 2.6 | Yes | No | NALCN | NM_052867.2 | Hypotonia, infantile, with psychomotor retardation and characteristic facies(OMIM:615419) | AR | hom | Nonsense | c.4150C>T | p.(R1384*) | Inherited from parents | LP | Delayed speech and language development,Muscular hypotonia,Motor delay,Central hypotonia |
| 00080953 | SA | f | 3.2 | No | Yes | TRDN | NM_006073.3 | Ventricular tachycardia, catecholaminergic polymorphic, 5, with or without muscle weakness(OMIM:615441) | AR | hom | Frameshift | c.438_442del | p.(K147*) | Inherited from parents | LP | Muscular hypotonia,Hyporeflexia,Motor delay,Feeding difficulties |
| 00080954 | ME | f | 2.2 | No | Yes | SLC19A3 | NM_025243.3 | Thiamine metabolism dysfunction syndrome 2 (biotin- or thiamine-responsive encephalopathy type 2)(OMIM:607483) | AR | hom | Missense | c.1264A>G | p.(T422A) | Inherited from parents | P | Abnormality of the kidney,Microcephaly,Abnormality of the face,Long philtrum,Micrognathia,Cataract,Long eyelashes,Abnormality of the endocrine system,Global developmental delay,Motor delay,Hypertonia,Failure to thrive,Intrauterine growth retardation,Decreased fetal movement,Oligohydramnios,Atria septal defect,Coarctation of aorta,Gastroesophageal reflux,Hiatus hernia,Respiratory distress,Abnormality of the cerebral white matter,Multiple joint contractures,Decreased body weight,Abnormality of the cheek,Depressed nasal bridge,Corpus callosum atrophy,External ear malformation,Abnormality of the hairline,Delayed myelination |
| 00080955 | SA | u | unknown | Yes | Yes | TRIOBP | NM_001039141.2 | Deafness, autosomal recessive 28(OMIM:609823) | AR | hom | Nonsense | c.2320C>T | p.(R774*) | Inherited from parents | LP | Hearing impairment |
| 00080956 | ME | f | 2.5 | Yes | No | ADNP | NM_015339.2 | Helsmoortel-van der Aa syndrome(OMIM:615873) | AD | het | Frameshift | c.2157del | p.(Y719*) | De Novo | LP | Macrocephaly,Abnormality of the cerebral white matter |
| 00080957 | ME | m | 3.4 | Yes | Yes | FBXL4 | NM_012160.4 | m DNA depletion syndrome 13 (encephalomyopathic type)(OMIM:615471) | AR | hom | Nonsense | c.292C>T | p.(R98*) | Inherited from parents | LP | Macrocephaly,Abnormality of the face,Global developmental delay,Metabolic acidosis,Abnormality of the periventricular white matter,Lactic acidosis,CNS hypomyelination,Cerebellar dysplasia,Infantile axial hypotonia,Abnormality of pyruvate family amino acid metabolism |
| 00080958 | ME | f | 3.9 | Yes | Yes | PGAP3 | NM_033419.4 | Hyperphosphatasia with mental retardation syndrome 4(OMIM:615716) | AR | hom | Nonsense | c.924C>A | p.(Y308*) | Inherited from parents | LP | Abnormality of the face,Coarse facial features,Broad face,Abnormality of the maxilla,Short neck,Upslanted palpebral fissure,Synophrys,Autism,Delayed speech and language development,Hirsutism,Intellectual disability,Global developmental delay,Motor delay,Generalized hypotonia,Reduced tendon reflexes,Cerebellar vermis hypoplasia,Ventricular septal defect,Delayed gross motor development,Poor head control,Abnormal head movements,Abnormality of the cerebral white matter,Periventricular cysts,Prominent epicanthal folds,Intellectual disability, severe,Abnormality of mouth shape,Stereotypical hand wringing,Widened subarachnoid space,Abnormality of movement |
| 00080959 | ME | m | 13.5 | Yes | Yes | OXCT1 | NM_000436.3 | Succinyl CoA:3-oxoacid CoA transferase deficiency (OMIM:245050) | AR | hom | Missense | c.1402C>T | p.(R468C) | Inherited from parents | P | Hypoglycemia,Lactic acidosis,Hyperalaninemia |
| 00080960 | ME | f | 3.0 | Yes | Yes | ROBO3 | NM_022370.3 | Gaze palsy, horizontal, with progressive scoliosis (OMIM:607313) | AR | hom | Nonsense | c.3937C>T | p.(Q1313*) | Inherited from parents | LP | Torticollis,Ophthalmoplegia,Oculomotor apraxia,Hypertonia |
| 00080961 | ME | m | 3.7 | Yes | N.i | SGCA | NM_000023.2 | Muscular dystrophy, limb-girdle, type 2D (OMIM:608099) | AR | hom | Missense | c.739G>A | p.(V247M) | Inherited from parents | P | Seizures,Abnormality of the mitral valve,Mitral valve prolapse,Abnormality of the left ventricle,Ketosis,Abnormality of cardiac atrium,Abnormality of acetylcarnitine metabolism,Abnormal levels of creatine kinase in blood |
| 00080962 | ME | f | 6.5 | Yes | No | ADCK4 | NM_024876.3 | Nephrotic syndrome, type 9(OMIM:615573) | AR | hom | Missense | c.532C>T | p.(R178W) | Inherited from parents | P | Proteinuria,Dilated cardiomyopathy,Prolonged QT interval,Cardiac arrest,Cerebral ischemia,Low-molecular-weight proteinuria,Stage 5 chronic kidney disease,Subcortical white matter calcifications,Congenital nephrotic syndrome |
| 00080963 | ME | m | 7.6 | Yes | Yes | L2HGDH | NM_024884.2 | L-2-hydroxyglutaric aciduria(OMIM:236792) | AR | hom | Frameshift | c.1015del | p.(R339Dfs*13) | Inherited from parents | P | Macrocephaly,Ataxia,Motor delay,Abnormality of the globus pallidus,Abnormality of the cerebral white matter,Abnormality of movement,Abnormality of the dentate nucleus |
| 00080964 | ME | m | 1.9 | No | N.i | SGCA | NM_000023.2 | Muscular dystrophy, limb-girdle, type 2D (OMIM:608099) | AR | hom | Missense | c.739G>A | p.(Vl247M) | Inherited from parents | P | Proteinuria,Focal segmental glomerulosclerosis,Enlarged kidneys,Facial edema,Edema,Ascites,Increased circulating very-low-density lipoprotein cholesterol,Hyperechogenic kidneys,Steroid-resistant nephrotic syndrome |
| 00080965 | ME | m | 5.3 | No | No | GATAD2B | NM_020699.2 | Mental retardation, autosomal dominant 18 (OMIM:615074) | AD | het | Frameshift | c.815_816insTCCA | p.(Q272Hfs*11) | De Novo | LP | Strabismus,Delayed speech and language development,Global developmental delay,Motor delay,Hyperreflexia,Abnormal delivery,Premature rupture of membranes,Morphological abnormality of the central nervous system,Gait ataxia,Generalized tonic-clonic seizures,Unsteady gait,EEG abnormality,Febrile seizures,Congenital encephalopathy,Muscular hypotonia of the trunk,Abnormality of the palmar creases,Feeding difficulties |
| 00080966 | ME | f | 3.2 | No | No | MECP2 | NM_001110792.1 | Rett syndrome(OMIM:312750) | X-linked | het | Nonsense | c.916C>T | p.(R306*) | De Novo | P | Delayed speech and language development,Syndactyly,Intellectual disability,Seizures,Muscular hypotonia,Motor delay,Muscle weakness,Joint hypermobility,Obesity,Overgrowth,Developmental regression,Lactic acidosis,Increased serum pyruvate,Dilatation of the renal pelvis,Clinodactyly |
| 00080967 | ME | m | 6.7 | Yes | No | ADCK3 | NM_020247.4 | Coenzyme Q10 deficiency, primary, 4 (OMIM:612016) | AR | hom | Missense | c.814G>T | p.(G272C) | Inherited from parents | LP | Oculomotor apraxia,Delayed speech and language development,Ataxia,Global developmental delay,Cerebellar atrophy,Gait disturbance,Breech presentation,Frequent falls,Neonatal respiratory distress |
| 00080968 | ME | m | 11.9 | No | No | EFEMP2 | NM_016938.4 | Cutis laxa, autosomal recessive, type IB(OMIM:614437) | AR | hom | Missense | c.481G>A | p.(E161K) | Inherited from parents | P | Ureterocele,Abnormality of the pinna,Short neck,Chorioretinal coloboma,Abnormality of the aortic valve,Aortic regurgitation,Secundum atrial septal defect,Aortic dilatation,Abnormal facial shape,Hypoplastic nipples,Ascending aortic aneurysm,Dilatation of the ascending aorta,Thick nasal septum,Arrhythmia,Heart murmur,Abnormality of limbs,Angioedema |
| 00080969 | Eu | m | 4.1 | No | No | MBD5 | NM_018328.4 | Mental retardation, autosomal dominant 1(OMIM:156200) | AD | het | Frameshift | c.288_301del | p.(K98*) | De Novo | LP | Behavioral abnormality,Autism,Delayed speech and language development,Hyperextensible skin,Abnormality of skin pigmentation,Intellectual disability,Ataxia,Motor delay,Muscle weakness,Hepatosplenomegaly,Growth delay,Ketosis,Organic aciduria,Abnormal facial shape,Lactic acidosis,Abnormality of the mitochondrion,Brain atrophy,Neurodevelopmental delay |
| 00080970 | ME | u | unknown | No | No | JAG1 | NM_000214.2 | Alagille syndrome (OMIM:118450) | AD | het | Nonsense | c.2566C>T | p.(Q856*) | De Novo | P | Deeply set eye,Biliary tract abnormality,Cholestasis,Frontal bossing,Prolonged neonatal jaundice |
| 00080971 | ME | f | 0.5 | Yes | No | TMPRSS15 | NM_002772.2 | Enterokinase deficiency(OMIM:226200) | AR | hom | Nonsense | c.2135C>G | p.(S712*) | Inherited from parents | P | Irritability,Jaundice,Spasticity,Hypertonia,Abnormality of the liver,Failure to thrive,Anemia,Diarrhea,Elevated hepatic transaminases,Hypoalbuminemia,Lactic acidosis,Abnormality of the coagulation cascade,Muscle stiffness,Lacticaciduria,Prolonged prothrombin time,Edema of the lower limbs,Abnormal hemoglobin,Feeding difficulties,Abnormal levels of creatine kinase in blood,Sepsis |
| 00080972 | ME | f | 6.9 | Yes | Yes | HADHB | NM_000183.2 | Trifunctional protein deficiency(OMIM:609015) | AR | hom | Splicing | c.631-1G>A | p.? | Inherited from parents | LP | Metabolic acidosis,Neonatal death,Severe lactic acidosis,Mildly elevated creatine phosphokinase,Long chain 3 hydroxyacyl coA dehydrogenase deficiency |
| 00080973 | ME | m | 1.9 | Yes | No | SAMHD1 | NM_015474.3 | Aicardi-Goutieres syndrome 5(OMIM:612952) | AR | hom | Splicing | c.625+1G>A | p.? | Inherited from parents | LP | Irritability,Anxiety,Spasticity,Global developmental delay,Motor delay,Hypertonia,Brisk reflexes,Developmental regression,Babinski sign,Central hypotonia |
| 00080974 | ME | f | 7.9 | No | Yes | POMT1 | NM_007171.3 | Muscular dystrophy-dystroglycanopathy (congenital with mental retardation), type B, 1(OMIM:613155) | AR | hom | Frameshift | c.2179_2180del | p.(S727Afs*3) | Inherited from parents | P | Abnormality of the anterior fontanelle,Microcephaly,Hypotelorism,Intellectual disability,Seizures,Muscular hypotonia,Motor delay,Generalized hypotonia,Dandy-Walker malformation,Cerebellar vermis hypoplasia,Cerebellar hypoplasia,Hyperreflexia,Polymicrogyria,Delayed gross motor development,EEG abnormality,Primitive reflexes (palmomental, snout, glabellar),Short stature,Severely dysplastic cerebellum,Severe global developmental delay,Central hypotonia,Cerebellar hemisphere hypoplasia |
| 00080975 | ME | m | 3.1 | Yes | Yes | ASNS | NM_183356.3 | Asparagine synthetase deficiency(OMIM:615574) | AR | hom | Missense | c.1193A>G | p.(Y398C) | N.i | P | Microcephaly,Narrow forehead,Intellectual disability,Seizures,Spasticity,Cerebellar hypoplasia,Respiratory distress,Clonus,Lower limb hyperreflexia,Severe global developmental delay |
| 00080976 | ME | m | 8.6 | No | No | ARID1B | NM_020732.3 | Mental retardation, autosomal dominant 12(OMIM:614562) | AD | het | Frameshift | c.5570_5573del | p.(K1857Sfs*17) | De Novo | LP | Abnormality of the face,Myopia,Intellectual disability,Seizures,Global developmental delay,Attention deficit hyperactivity disorder |
| 00080977 | ME | f | 4.8 | Yes | No | KIF1A | NM_001244008.1 | Mental retardation, autosomal dominant 9(OMIM:614255) | AD | het | Missense | c.647G>A | p.(R216H) | De Novo | LP | Renal cyst,Abnormality of the face,Microretrognathia,Hypoplasia of the maxilla,Flared nostrils,Short neck,Strabismus,Abnormality of the eyelid,Downslanted palpebral fissures,Ptosis,Blue sclerae,Narrow chest,Jaundice,Hirsutism,Thickened skin,Muscular hypotonia,Global developmental delay,Motor delay,Brisk reflexes,Polyhydramnios,Redundant skin,Ventricular septal defect,Pulmonic stenosis,Dilated cardiomyopathy,Tachycardia,Abnormality of the tricuspid valve,Spina bifida,obsolete Malformation of the heart and great vessels,Tachypnea,Lower limb hypertonia,Equinovarus deformity,Edema of the lower limbs,Edema of the upper limbs,Sinus venosus atrial septal defect,Abnormality of the aortic arch,Abnormality of the supraorbital ridges |
| 00080978 | ME | f | 2.9 | Yes | Yes | GAMT | NM_000156.5 | Cerebral creatine deficiency syndrome 2(OMIM:612736) | AR | hom | Frameshift | c.609dup | p.(R204Efs*63) | Inherited from parents | LP | Delayed speech and language development,Intellectual disability,Global developmental delay,Motor delay,Focal T2 hyperintense basal ganglia lesion |
| 00080979 | ME | m | 1.0 | No | N.i | COL4A1 | NM_001845.4 | Porencephaly 1(OMIM:175780) | AD | het | Missense | c.3715G>A | p.(G1239R) | De novo | P | Microcephaly,Ptosis,Congenital cataract,Myopia,Esotropia,Delayed speech and language development,Spasticity,Global developmental delay,Motor delay,Hypertonia,Encephalopathy,Stroke-like episodes,Cerebral calcification,Cerebellar hemorrhage,Brain atrophy,Delayed myelination,Neonatal asphyxia |
| 00080980 | ME | m | 17.1 | Yes | Yes | ADAT3 | NM_138422.2 | Mental retardation, autosomal recessive 36(OMIM:615286) | AR | hom | Missense | c.430G>A | p.(V144M) | Inherited from parents | P | Restlessness,Intellectual disability,Muscular hypotonia,Global developmental delay,Gait disturbance,Limb joint contracture,Short stature,Decreased body weight |
| 00080981 | ME | f | 8.8 | Yes | No | PC | NM_001040716.1 | Pyruvate carboxylase deficiency(OMIM:266150) | AR | hom | Missense | c.2525G>A | p.(R842Q) | Inherited from parents | LP | Delayed speech and language development,Muscular hypotonia,Motor delay,Stroke,Encephalopathy,Metabolic acidosis,Hyperammonemia,Generalized seizures,Neonatal respiratory distress,Lactic acidosis,Intellectual disability, severe,Severe global developmental delay,m respiratory chain defects |
| 00080982 | ME | m | 11.5 | N.i | N.i | CLN6 | NM_017882.2 | Ceroid lipofuscinosis, neuronal, 6(OMIM:601780) | AR | hom | In-frame | c.1_11del | p.? | Inherited from parents | LP | Generalized seizures,Developmental regression |
| 00080983 | ME | m | 0.7 | No | No | NDUFS4 | NM_002495.2 | Leigh syndrome(OMIM:256000) | AR | hom | Frameshift | c.462del | p.(K154Nfs*35) | Inherited from parents | P | Ophthalmoplegia,Motor delay,Muscle weakness,Failure to thrive,Chorea,Increased CSF lactate,Abnormality of m metabolism,m myopathy,Focal T2 hyperintense brainstem lesion |
| 00080984 | ME | f | 0.8 | Yes | Yes | ALDH7A1 | NM_001182.4 | Epilepsy, pyridoxine-dependent(OMIM:266100) | AR | hom | Frameshift | c.1431dup | p.(C478Lfs*14) | Inherited from parents | LP | Jaundice,Seizures,Motor delay,Agenesis of corpus callosum,Muscle weakness,Dystonia,Developmental regression,Increased CSF lactate,Hyperalaninemia,Hypointensity of cerebral white matter on MRI,Periventricular cysts,Postnatal growth retardation,Abnormality of the internal capsule,Abnormal basal ganglia MRI signal intensity,Abnormality of movement |
| 00080985 | ME | m | 3.6 | Yes | Yes | CLP1 | NM_006831.2 | Pontocerebellar hypoplasia, type 10(OMIM:615803) | AR | hom | Missense | c.419G>A | p.(R140H) | Inherited from parents | P | Microcephaly,Delayed speech and language development,Intellectual disability,Motor delay |
| 00080986 | ME | f | 6.7 | N.i | N.i | DLD | NM_000108.3 | Dihydrolipoamide dehydrogenase deficiency(OMIM:246900) | AR | hom | Missense | c.685G>T | p.(G229C) | N.i | P | Metabolic acidosis,Recurrent hypoglycemia,Vomiting |
| 00080987 | ME | f | 5.5 | Yes | No | TERT | NM_198253.2 | Dyskeratosis congenita, autosomal recessive 4(OMIM:613989) | AR | hom | Missense | c.604G>A | p.(A202T) | Inherited from parents | P | Oligodontia,Abnormality of the fingernails,Hidrotic ectodermal dysplasia,Sparse hair,Dystrophic fingernails |
| 00080988 | ME | m | 1.2 | No | Yes | DYRK1A | NM_001396.3 | Mental retardation, autosomal dominant 7(OMIM:614104) | AD | het | Missense | c.1040T>G | p.(L347R) | De Novo | LP | Microcephaly,Intellectual disability,Seizures,Spasticity,Global developmental delay,Motor delay,Hypertonia,Abnormal facial shape,Cortical dysplasia,Short stature,Cortical gyral simplification |
| 00080989 | ME | f | 2.4 | Yes | No | FBXL4 | NM_012160.4 | m DNA depletion syndrome 13 (encephalomyopathic type)(OMIM:615471) | AR | hom | Nonsense | c.292C>T | p.(R98*) | Inherited from parents | LP | Microcephaly,Muscular hypotonia,Lethargy,Absent speech,Intrauterine growth retardation,Metabolic acidosis,Hyperammonemia,Diarrhea,Abnormality of the basal ganglia,Lactic acidosis,Short stature,Acute encephalopathy,Severe global developmental delay,Feeding difficulties |
| 00080990 | ME | m | 2.3 | N.i | N.i | KCNA2 | NM_004974.3 | Epileptic encephalopathy, early infantile, 32(OMIM:616366) | AD | het | Missense | c.1120A>G | p.(T374A) | De Novo | LP | Microcephaly,Delayed speech and language development,Intellectual disability,Seizures,Global developmental delay,Motor delay,Abnormality of movement |
| 00080991 | ME | f | 14.0 | Yes | No | SMC1A | NM_006306.3 | Cornelia de Lange syndrome 2(OMIM:300590) | X | het | Nonsense | c.2897C>G | p.(S966*) | De Novo | LP | Self-mutilation,Delayed speech and language development,Intellectual disability,Seizures,Motor delay,Generalized seizures |
| 00080992 | Eu | f | 6.6 | N.i | No | GRIN2B | NM_000834.3 | Mental retardation, autosomal dominant 6(OMIM:613970) | AD | het | Nonsense | c.737C>A | p.(S246*) | De Novo | LP | Glaucoma,Behavioral abnormality,Autism,Dementia,Delayed speech and language development,Diabetes mellitus,Intellectual disability,Muscular hypotonia,Motor delay,Areflexia,Muscle weakness,EEG abnormality,Abnormality of connective tissue,Ventricular extrasystoles,Hypoplasia of the frontal lobes,Arrhythmia,Abnormal fear/anxiety-related behavior |
| 00080993 | Eu | f | 0.3 | N.i | No | KAT6B | NM_012330.3 | Genitopatellar syndrome(OMIM:606170) | AD | het | Nonsense | c.4096G>T | p.(E1366*) | De Novo | LP | Cleft palate,Bifid uvula,Abnormality of the chin,Abnormality of the pinna,Prominent antihelix,Abnormality of the nasal bridge,Wide nasal bridge,Choanal stenosis,Agenesis of corpus callosum,Ventricular septal defect,Talipes equinovarus,Abnormal facial shape,Respiratory distress,Apnea,Respiratory difficulties,Absent brainstem auditory responses,Hypoplastic superior helix,Abnormality of the antihelix,Feeding difficulties |
| 00080994 | ME | f | 5.7 | Yes | No | CLN6 | NM_017882.2 | Ceroid lipofuscinosis, neuronal, 6(OMIM:601780) | AR | hom | In-frame | c.794_796del | p.(S265del) | Inherited from parents | P | Optic atrophy,Seizures,Spasticity,Hypertonia,Encephalopathy,Developmental regression,Leukodystrophy,Abnormality of the cerebral white matter,Progressive inability to walk,Loss of ability to walk |
| 00080995 | ME | f | 1.2 | N.i | N.i | CBL | NM_005188.3 | Noonan syndrome-like disorder with or without juvenile myelomonocytic leukemia(OMIM:613563) | AD | het | Splicing | c.1227+5_1227+8del | p.? | De Novo | LP | Microcephaly,Aplasia cutis congenita,Atria septal defect,Abnormality of toe,Short phalanx of finger |
| 00080996 | Eu | m | 0.6 | Yes | Yes | TJP2 | NM_001170416.1 | Cholestasis, progressive familial intrahepatic 4 (OMIM:615878) | AR | hom | Nonsense | c.1093C>T | p.(R365*) | Inherited from parents | LP | Cholestasis,Hepatic failure,Intrahepatic cholestasis |
| 00080997 | ME | f | 13.1 | Yes | No | ATP6V0A4 | NM_020632.2 | Renal tubular acidosis, distal, autosomal recessive(OMIM:602722) | AR | hom | Splicing | c.292-1G>A | p.? | Inherited from parents | P | Proteinuria,Nephrocalcinosis,Hearing impairment,Progressive sensorineural hearing impairment,Hypothyroidism,Distal renal tubular acidosis |
| 00080998 | Eu | m | 6.2 | No | N.i | GRIN2B | NM_000834.3 | Mental retardation, autosomal dominant 6(OMIM:613970) | AD | het | Missense | c.2252T>C | p.(I751T) | De Novo | LP | Delayed speech and language development,Intellectual disability,Muscular hypotonia,Motor delay,Joint hypermobility,Abnormality of the hair |
| 00080999 | SA | u | unknown | No | N.i | UBE3A | NM_000462.3 | Angelman syndrome(OMIM:105830) | AD | het | Frameshift | c.2572_2575dup | p.(K859Tfs*2) | De Novo | P | Microcephaly,Behavioral abnormality,Seizures,Global developmental delay,Brisk reflexes,Happy demeanor |
| 00081000 | ME | m | 3.3 | No | No | ZEB2 | NM_014795.3 | Mowat-Wilson syndrome(OMIM:235730) | AD | het | Nonsense | c.2769C>A | p.(Y923*) | De Novo | LP | Delayed speech and language development,Seizures,Motor delay,Agenesis of corpus callosum |
| 00081001 | Eu | m | 3.0 | No | N.i | ASPM | NM_018136.4 | Microcephaly 5, primary, autosomal recessive(OMIM:608716) | AR | hom | Nonsense | c.7323T>A | p.(Y2441*) | Inherited from parents | LP | Microcephaly,Delayed speech and language development,Intellectual disability,Seizures,Global developmental delay,Polymicrogyria,Abnormal cortical gyration |
| 00081002 | ME | u | unknown | N.i | No | CD59 | NM_203330.2 | Hemolytic anemia, CD59-mediated, with or without immune-mediated polyneuropathy(OMIM:612300) | AR | hom | Nonsense | c.323C>A | p.(S108*) | Inherited from parents | LP | Facial edema,Intellectual disability,Seizures,Lethargy,Motor delay,Encephalopathy,Reduced tendon reflexes,Slurred speech,Gliosis,Abnormal pattern of respiration,Generalized muscle weakness,Peripheral axonal neuropathy,Proximal muscle weakness,Cerebellar cortical atrophy,Muscle flaccidity,Facial palsy,Abnormal myelination,Abnormality of movement |
| 00081003 | ME | f | 14.1 | Yes | Yes | PPOX | NM_001122764.1 | Porphyria variegata(OMIM:176200) | AR | hom | In-frame | c.1108_1119del | p.(G370_W373del) | Inherited from parents | LP | Nystagmus,Hypopigmentation of the skin,Seizures,Leukodystrophy,Ichthyosis,Primary adrenal insufficiency,Abnormality of the heme biosynthetic pathway,Neonatal asphyxia,Inappropriate crying |
| 00081004 | ME | m | 10.3 | Yes | No | SPATA7 | NM_018418.4 | Leber congenital amaurosis 3(OMIM:604232) | AR | hom | Frameshift | c.296_297del | p.(E99Vfs*5) | Inherited from parents | LP | Visual impairment,Leber optic atrophy |
| 00081005 | ME | f | 0.3 | Yes | No | CFL2 | NM_138638.4 | Nemaline myopathy 7, autosomal recessive(OMIM:610687) | AR | hom | Frameshift | c.338del | p.(S113Tfs*3) | Inherited from parents | LP | Hyperthyroidism,Muscular hypotonia,Lethargy,Reduced tendon reflexes,Failure to thrive,Polyhydramnios,Cardiomegaly,Respiratory insufficiency,Neonatal breathing dysregulation,Muscle flaccidity,Ventouse delivery,Cephalohematoma,Neonatal asphyxia,Subdural hemorrhage |
| 00081006 | ME | m | 3.5 | No | No | CTNNB1 | NM_001098210.1 | Mental retardation, autosomal dominant 19(OMIM:615075) | AD | het | Nonsense | c.1981C>T | p.(R661*) | De Novo | LP | Microcephaly,Micrognathia,Delayed speech and language development,Global developmental delay,Abnormal facial shape,Lower limb spasticity,Central hypotonia |
| 00081007 | ME | f | 0.9 | No | N.i | RAF1 | NM_002880.3 | Noonan syndrome 5(OMIM:611553) | AD | het | Missense | c.770C>T | p.(S257L) | De Novo | P | Coarse facial features,Cystic hygroma,Global developmental delay,Redundant skin,Hoarse voice,Mitral regurgitation,Subaortic stenosis,Left ventricular hypertrophy,Short stature,Relative macrocephaly,Brain atrophy |
| 00081008 | ME | m | 0.5 | Yes | No | IGHMBP2 | NM_002180.2 | Neuronopathy, distal hereditary motor, type VI(OMIM:604320) | AR | hom | Nonsense | c.1813C>T | p.(R605*) | Inherited from parents | P | Muscular hypotonia,Respiratory failure requiring assisted ventilation,Coarctation of abdominal aorta |
| 00081009 | ME | f | 3.5 | Yes | Yes | PCCA | NM_000282.3 | Propionicacidemia(OMIM:606054) | AR | hom | Missense | c.425G>A | p.(G142D) | Inherited from parents | P | Microcephaly,Muscular hypotonia,Global developmental delay,Propionicacidemia,Short stature,Abnormality of body weight |
| 00081010 | Eu | m | 14.6 | N.i | N.i | KCNA1 | NM_000217.2 | Episodic ataxia/myokymia syndrome(OMIM:160120) | AD | het | Missense | c.1007G>A | p.(G336E) | De Novo | LP | Seizures,Generalized seizures,Paroxysmal dyskinesia,Focal seizures |
| 00081011 | Eu | m | 1.4 | N.i | N.i | PTPN11 | NM_002834.3 | Noonan syndrome 1(OMIM:163950) | AD | het | Missense | c.854T>C | p.(F285S) | De Novo | P | Protruding ear,Proptosis,Muscular hypotonia,Dandy-Walker malformation,Pulmonic stenosis,Abnormal facial shape |
| 00081012 | ME | f | 1.9 | Yes | N.i | ANTXR2 | NM_001145794.1 | Hyaline fibromatosis syndrome(OMIM:228600) | AR | hom | Missense | c.134T>C | p.(L45P) | N.i | P | Muscular hypotonia,Abnormal facial shape,Distal arthrogryposis |
| 00081013 | Eu | m | 5.3 | No | No | STXBP1 | NM_003165.3 | Epileptic encephalopathy, early infantile, 4(OMIM:612164) | AD | het | Nonsense | c.703C>T | p.(R235*) | De Novo | P | Tall stature,Astigmatism,Visual impairment,Myopia,Behavioral abnormality,Autism,Delayed speech and language development,Seizures,Ataxia,Global developmental delay,Cerebellar hypoplasia,Failure to thrive,Generalized seizures,EEG abnormality,Developmental regression,Focal seizures,Abnormality of movement |
| 00081014 | ME | u | unknown | Yes | N.i | CRTAP | NM_006371.4 | Osteogenesis imperfecta, type VII(OMIM:610682) | AR | hom | Frameshift | c.160_167del | p.(K54Rfs*104) | Inherited from parents | LP | Abnormality of the skeletal system,Abnormality of limbs |
| 00081015 | ME | m | 2.1 | Yes | Yes | HEXB | NM_000521.3 | Sandhoff disease, infantile, juvenile, and adult forms(OMIM:268800) | AR | hom | Splicing | c.1082+5G>A | p.? | Inherited from parents | P | Delayed speech and language development,Intellectual disability,Muscular hypotonia,Motor delay,Encephalopathy,Generalized seizures,Developmental regression,Focal seizures,Brain atrophy |
| 00081016 | ME | u | unknown | No | Yes | KMT2A | NM_001197104.1 | Wiedemann-Steiner syndrome(OMIM:605130) | AD | het | Nonsense | c.10780C>T | p.(Q3594*) | De Novo | LP | Epicanthus,Hypertelorism,Delayed speech and language development,Hypothyroidism,Sacral dimple,Hirsutism,Seizures,Muscular hypotonia,Global developmental delay,Umbilical hernia,Abnormal facial shape,Periventricular leukomalacia |
| 00081017 | ME | u | unknown | Yes | Yes | ABCA3 | NM_001089.2 | Surfactant metabolism dysfunction, pulmonary, 3(OMIM:610921) | AR | hom | Missense | c.604G>A | p.(G202R) | Inherited from parents | LP | Abnormality of the lung,Respiratory distress,Respiratory failure requiring assisted ventilation |
| 00081018 | Eu | f | 2.5 | No | No | SLC13A5 | NM_177550.4 | Epileptic encephalopathy, early infantile, 25(OMIM:615905) | AR | hom | Missense | c.655G>A | p.(G219R) | Inherited from parents | P | Delayed speech and language development,Ataxia,Motor delay,Generalized seizures |
| 00081019 | ME | u | unknown | Yes | Yes | PEX1 | NM_000466.2 | Peroxisome biogenesis disorder 1A (Zellweger)(OMIM:214100) | AR | hom | Nonsense | c.2875C>T | p.(R959*) | Inherited from parents | LP | Cryptorchidism,Renal cyst,Hydronephrosis,Microcephaly,Hypertelorism,Visual impairment,Renal cortical cysts,Muscular hypotonia,Agenesis of corpus callosum,Patent ductus arteriosus,Patent foramen ovale,Talipes equinovarus,Abnormal facial shape,Respiratory distress,Generalized seizures,Tricuspid regurgitation,Dilation of lateral ventricles,Dilated third ventricle,Mixed demyelinating and axonal polyneuropathy,Abnormal myelination |
| 00081020 | SA | m | unknown | No | Yes | MYO15A | NM_016239.3 | Deafness, autosomal recessive 3(OMIM:600316) | AR | hom | Missense | c.8158G>C | p.(D2720H) | Inherited from parents | P | Hearing impairment |
| 00081021 | ME | m | 0.8 | Yes | Yes | ASAH1 | NM_004315.4 | Farber lipogranulomatosis(OMIM:228000) | AR | hom | Missense | c.1045C>G | p.(R349G) | Inherited from parents | P | Hydrocele testis,Microcephaly,Facial edema,Abnormality of the pinna,Anteverted nares,Short neck,Hepatosplenomegaly,Growth delay,Small for gestational age,Ascites,Premature birth,Atria septal defect,Patent ductus arteriosus,Mitral regurgitation,Pericardial effusion,Abnormality of the left ventricle,Nonimmune hydrops fetalis,Hypokinesia,Neonatal respiratory distress,Abnormality of the calvaria,Short stature,Hyperechogenic kidneys,Tricuspid regurgitation,Generalized edema,Incomplete cleft of the upper lip,Caesarian section,Subdural hemorrhage |
| 00081022 | ME | f | 3.7 | Yes | Yes | NTRK1 | NM_002529.3 | Insensitivity to pain, congenital, with anhidrosis(OMIM:256800) | AR | hom | Nonsense | c.526C>T | p.(Q176*) | Inherited from parents | P | Hyperactivity,Hypohidrosis,Muscular hypotonia,Global developmental delay,Fever,Unsteady gait,Sensory impairment,Decreased body weight |
| 00081023 | ME | u | unknown | Yes | Yes | ALMS1 | NM_015120.4 | Alstrom syndrome(OMIM:203800) | AR | hom | Nonsense | c.11291C>A | p.(S3764*) | Inherited from parents | LP | Microcephaly,Hearing impairment,Visual impairment,Spasticity,Cardiomyopathy,Dilated cardiomyopathy,Cardiac arrest,Brain atrophy |
| 00081024 | Eu | m | 0.9 | No | No | SLC16A2 | NM_006517.4 | Allan-Herndon-Dudley syndrome(OMIM:300523) | X | hemi | Nonsense | c.44G>A | p.(W15*) | Inherited from mother | LP | Visual impairment,Delayed speech and language development,Motor delay,Encephalopathy,Failure to thrive,Global brain atrophy,Developmental regression,Lactic acidosis,Increased serum pyruvate,Severe muscular hypotonia,Abnormal myelination |
| 00081025 | ME | m | 16.0 | Yes | No | AIPL1 | NM_014336.3 | Leber congenital amaurosis 4(OMIM:604393) | AR | hom | Frameshift | c.178dup | p.(H60Pfs*98) | Inherited from parents | LP | Visual loss,Nystagmus |
| 00081026 | ME | m | 2.0 | No | No | STXBP1 | NM_003165.3 | Epileptic encephalopathy, early infantile, 4(OMIM:612164) | AD | het | Missense | c.1060T>C | p.(C354R) | De Novo | P | Delayed speech and language development,Hyperextensible skin,Intellectual disability,Ataxia,Motor delay,Leukodystrophy,Rickets |
| 00081027 | ME | f | 0.3 | No | No | HRAS | NM_001130442.1 | Costello syndrome(OMIM:218040) | AD | het | Missense | c.34G>A | p.(G12S) | De Novo | P | Micrognathia,Low-set ears,Abnormality of the liver,Polyhydramnios,Premature birth,Abnormal facial shape,Frontal bossing,Abnormality of the intestine,Neonatal respiratory distress,Decreased body weight,Depressed nasal bridge,Caesarian section |
| 00081028 | ME | m | 3.1 | No | No | NF1 | NM_001042492.2 | Neurofibromatosis, type 1(OMIM:162200) | AD | het | Nonsense | c.5327C>A | p.(S1776*) | De Novo | LP | Coarse facial features,Glaucoma,Muscular hypotonia |
| 00081029 | ME | m | 1.3 | Yes | No | ALDOB | NM_000035.3 | Fructose intolerance(OMIM:229600) | AR | hom | Frameshift | c.360_363del | p.(N120Kfs*32) | Inherited from parents | P | Microcephaly,Anxiety,Delayed speech and language development,Jaundice,Global developmental delay,Motor delay,Cholestasis,Metabolic acidosis,Hepatomegaly,Short stature,Abnormality of body weight |
| 00081030 | SA | f | unknown | No | No | GPSM2 | NM_013296.4 | Chudley-McCullough syndrome(OMIM:604213) | AR | hom | Nonsense | c.379C>T | p.(R127*) | Inherited from parents | P | Hearing impairment |
| 00081031 | ME | f | 0.4 | Yes | N.i | DGUOK | NM_080916.1 | m DNA depletion syndrome 3 (hepatocerebral type)(OMIM:251880) | AR | hom | Frameshift | c.763_766dup | p.(F256*) | Inherited from parents | P | Microcephaly,Visual impairment,Nystagmus,Optic atrophy,Muscular hypotonia,Motor delay,Hepatic failure,Failure to thrive,Abnormality of coagulation,Abnormal facial shape,Increased serum lactate,Hepatomegaly,Lactic acidosis |
| 00081032 | ME | m | 13.8 | N.i | Yes | STAC3 | NM_145064.2 | Native American myopathy(OMIM:255995) | AR | hom | Missense | c.851G>C | p.(W284S) | N.i | P | Muscular hypotonia,Abnormal facial shape,Myopathic facies,Muscular dystrophy,Spinal deformities,Hand muscle atrophy,Type 1 muscle fiber atrophy |
| 00081033 | ME | m | 14.1 | Yes | Yes | PGAP1 | NM_024989.3 | Mental retardation, autosomal recessive 42(OMIM:615802) | AR | hom | Frameshift | c.2349delins TTGTTTGGGATTAT | p.(H783Qfs*2) | Inherited from parents | LP | Abnormality of the pinna,Autism,Delayed speech and language development,Abnormality of skin pigmentation,Intellectual disability,Seizures,Motor delay,Growth delay,Developmental regression,Clinodactyly of the 5th finger,Short stature,Decreased body weight,Shortening of all distal phalanges of the fingers,Tapered distal phalanges of finger |
| 00081034 | SA | f | unknown | Yes | Yes | PCDH15 | NM_001142763.1 | Deafness, autosomal recessive 23(OMIM:609533) | AR | hom | Splicing | c.720+2T>C | p.? | Inherited from parents | LP | Hearing impairment |
| 00081035 | ME | m | 16.5 | Yes | No | PEX11B | NM_003846.2 | Peroxisome biogenesis disorder 14B(OMIM:614920) | AR | hom | Frameshift | c.148_149del | p.(S50Pfs*13) | Inherited from parents | LP | Congenital cataract,Autism,Delayed speech and language development,Seizures,Motor delay,Severe global developmental delay |
| 00081036 | ME | m | 1.0 | Yes | No | POMK | NM_032237.4 | Muscular dystrophy-dystroglycanopathy (congenital with brain and eye anomalies), type A, 12(OMIM:615249) | AR | hom | Nonsense | c.993C>G | p.(T331*) | Inherited from parents | LP | Hydrocephalus,Abnormality of the retina,Microcornea,Visual impairment,Cataract,Congenital cataract,Microphthalmia,Abnormality of the pupil,Muscular hypotonia,Motor delay,Cardiac shunt,Ventriculomegaly,Generalized seizures,Molar tooth sign on MRI,Abnormal cortical gyration,Decreased plasma carnitine,Abnormality of the pons,Bilateral microphthalmos,Anterior chamber synechiae,Persistent hyperplastic primary vitreous,Thickened cortex of bones |
| 00081037 | ME | m | 1.9 | No | No | HRAS | NM_001130442.1 | Costello syndrome(OMIM:218040) | AD | het | Missense | c.35G>C | p.(G12A) | De novo | P | Triangular face,Facial hemangioma,Micrognathia,Short neck,Prominent scalp veins,Global developmental delay,Failure to thrive,Laryngomalacia,Dilated cardiomyopathy,Mitral stenosis,Short toe,Abnormal facial shape,Frontal bossing,Lower limb spasticity,Delayed gross motor development,Pulmonary artery dilatation,Abnormality of the coronary arteries,Postnatal growth retardation,Short phalanx of finger,Stridor |
| 00081038 | ME | u | unknown | No | No | FBN1 | NM_000138.4 | Marfan syndrome(OMIM:154700) | AD | het | Nonsense | c.8275G>T | p.(E2759*) | De novo | LP | Wide mouth,Macrocephaly,Abnormality of the face,Retrognathia,Downslanted palpebral fissures,Proptosis,Myopia,Arachnodactyly,Long foot,Frontal bossing,Nail dystrophy,Thick vermilion border |
| 00081039 | ME | f | 5.5 | Yes | Yes | MMP13 | NM_002427.3 | Spahr type of metaphyseal dysplasia (OMIM:250400) | AR | hom | Missense | c.619T>G | p.(W207G) | Inherited from parents | P | Small for gestational age,Skeletal dysplasia,Bowing of the legs,Abnormality of the humeral metaphyses,Radial metaphyseal irregularity,Short stature,Decreased body weight,Abnormality of the tibial metaphysis,Metaphyseal dysplasia |
| 00081040 | ME | u | unknown | Yes | Yes | GCDH | NM_000159.2 | Glutaricaciduria, type I(OMIM:231670) | AR | hom | Missense | c.1063C>T | p.(R355C) | Inherited from parents | P | Delayed speech and language development,Intellectual disability,Motor delay,Growth delay,Organic aciduria,Developmental regression,Abnormality of the cerebral white matter,Recurrent infections,Focal seizures,Atrophy/Degeneration involving the caudate nucleus,Delayed myelination,Widened subarachnoid space,Punctate periventricular T2 hyperintense foci |
| 00081041 | Eu | f | 4.2 | No | No | FOXG1 | NM_005249.4 | Rett syndrome, congenital variant (OMIM:613454) | AD | het | Nonsense | c.385G>T | p.(E129*) | De Novo | LP | Microcephaly,Global developmental delay,Pachygyria,Abnormality of extrapyramidal motor function,Generalized seizures,Cerebral palsy,Abnormality of movement |
| 00081042 | Oceania | f | 0.7 | No | No | DNM1L | NM_012062.4 | Encephalopahty, lethal, due to defective m peroxisomal fission(OMIM:614388) | AD | het | Missense | c.607G>A | p.(V203I) | De Novo | LP | Nystagmus,Delayed speech and language development,Spasticity,Global developmental delay,Areflexia,Tongue fasciculations,Dystonia,Craniosynostosis,Chorea,Recurrent respiratory infections,Peripheral neuropathy,Feeding difficulties,Abnormal myelination,Cerebral white matter atrophy,Bilateral vocal cord paralysis |
| 00081043 | ME | m | 0.2 | Yes | Yes | TJP2 | NM_001170416.1 | Cholestasis, progressive familial intrahepatic 4(OMIM:615878) | AR | hom | Nonsense | c.1093C>T | p.(R365*) | Inherited from parents | LP | Depressed nasal ridge,Jaundice,Abnormal facial shape,Absent eyebrow,Elevated hepatic transaminases |
| 00081044 | ME | f | 8.4 | Yes | Yes | CBS | NM_001178008.1 | Thrombosis, hyperhomocysteinemic(OMIM:236200) | AR | hom | Missense | c.1039G>A | p.(G347S) | Inherited from parents | P | Behavioral abnormality,Lens subluxation,Arachnodactyly,Intellectual disability,Muscular hypotonia,Specific learning disability,Ascites,Weight loss,Abnormal thrombosis,Abnormal facial shape,Diarrhea,Abdominal pain,Homocystinuria,Abnormality of the cerebral white matter,Multiple joint contractures,Cachexia,Abnormality of the gallbladder,Abnormality of methionine metabolism |
| 00081045 | SA | m | 3.1 | Yes | N.i | COLQ | NM_005677.3 | Myasthenic syndrome, congenital, 5(OMIM:603034) | AR | hom | Splicing | c.955-2A>C | p.? | Inherited from parents | LP | Anophthalmia,Muscle weakness,Weak voice,Respiratory failure,Progressive ptosis |
| 00081046 | ME | f | 8.9 | No | Yes | L2HGDH | NM_024884.2 | L-2-hydroxyglutaric aciduria(OMIM:236792) | AR | hom | Missense | c.1319C>A | p.(S440Y) | Inherited from parents | LP | Macrocephaly,Torticollis,Strabismus,Seizures,Global developmental delay,Hemiparesis,Specific learning disability,Fever,Abnormality of the caudate nucleus,Abnormality of brainstem morphology,Abnormality of the globus pallidus,Cortical dysplasia,Dicarboxylic aciduria,Acute encephalopathy,Diffuse white matter abnormalities,L-2-hydroxyglutaric aciduria |
| 00081047 | ME | m | 2.9 | Yes | No | FKRP | NM_001039885.2 | Muscular dystrophy-dystroglycanopathy (congenital with or without mental retardation), type B, 5(OMIM:606612) | AR | hom | Missense | c.1364C>A | p.(A455D) | Inherited from parents | P | Seizures,Developmental regression,Leukodystrophy,Abnormal pyramidal signs |
| 00081048 | ME | m | 0.8 | Yes | No | KCNQ2 | NM_172107.2 | Seizures, benign neonatal, 1 (OMIM:121200) | AD | het | Nonsense | c.1342C>T | p.(R448*) | De novo | P | Seizures |
| 00081049 | ME | m | 12.1 | Yes | Yes | DEAF1 | NM_021008.3 | Mental retardation, autosomal dominant 24(OMIM:615828) | AR | hom | Splicing | c.997+4A>C | p.? | Inherited from parents | P | Autism,Stereotypic behavior,Self-mutilation,Global developmental delay,Abnormality of the corpus callosum,Leukodystrophy,Abnormality of the cerebral white matter,Dilation of lateral ventricles,Optic disc drusen |
| 00081050 | ME | m | 1.1 | Yes | Yes | ARL6IP1 | NM_015161.2 | Spastic paraplegia 61, autosomal recessive(OMIM:615685) | AR | hom | Nonsense | c.112C>T | p.(R38*) | Inherited from parents | LP | Intellectual disability,Spasticity,Motor delay,Agenesis of corpus callosum,Hypertonia,Developmental regression,Brain atrophy |
| 00081051 | Eu | u | unknown | Yes | Yes | HERC2 | NM_004667.5 | Mental retardation, autosomal recessive 38(OMIM:615516) | AR | hom | Splicing | c.4676-1G>A | p.? | Inherited from parents | LP | Seizures,Muscular hypotonia,Global developmental delay,Encephalopathy,Abnormality of the liver |
| 00081052 | ME | m | 1.8 | Yes | No | SLC13A5 | NM_177550.4 | Epileptic encephalopathy, early infantile, 25(OMIM:615905) | AR | hom | Frameshift | c.1227dup | p.(I410Hfs*13) | Inherited from parents | LP | Microcephaly,Mastoiditis,Delayed speech and language development,Seizures,Muscular hypotonia,Motor delay,Cerebellar atrophy,Failure to thrive,Subdural hemorrhage |
| 00081053 | ME | m | 10.4 | No | No | LIPH | NM_139248.2 | Woolly hair, autosomal recessive 2 with or without hypotrichosis(OMIM:604379) | AR | hom | In-frame | c.280_369dup | p.(G94_K123dup) | N.i | P | Curly hair,Generalized hypopigmentation of hair,Aplasia/Hypoplasia of the eyebrow |
| 00081054 | ME | f | 0.5 | N.i | N.i | UNC80 | NM_032504.1 | Hypotonia, infantile, with psychomotor retardation and characteristic facies 2(OMIM:616801) | AR | hom | Nonsense | c.151C>T | p.(R51*) | Inherited from parents | P | Intellectual disability,Global developmental delay,Generalized hypotonia,Failure to thrive,Diarrhea,Abdominal distention,Feeding difficulties |
| 00081055 | ME | f | 11.3 | No | No | NSD1 | NM_022455.4 | Sotos syndrome 1(OMIM:117550) | AD | het | Nonsense | c.1492C>T | p.(R498*) | De novo | P | Abnormality of the face,Blepharophimosis,Global developmental delay,Growth delay,Frontal bossing,Aplasia/Hypoplasia of the eyebrow |
| 00081056 | ME | u | unknown | No | N.i | MFSD8 | NM_152778.2 | Ceroid lipofuscinosis, neuronal, 7(OMIM:610951) | AR | hom | Splicing | c.863+1G>A | p.? | Inherited from parents | LP | Delayed speech and language development,Spasticity,Cerebellar atrophy,Hypertonia,Slurred speech,Small for gestational age,Generalized myoclonic seizures,Generalized seizures,Unsteady gait,EEG abnormality,Frequent falls,Abnormality of the periventricular white matter,Hyperactive deep tendon reflexes,Loss of ability to walk,Caesarian section,Abnormal brainstem MRI signal intensity |
| 00081057 | ME | m | 0.1 | Yes | Yes | SLC12A1 | NM_000338.2 | Bartter syndrome, type 1(OMIM:601678) | AR | hom | Frameshift | c.2952_2955del | p.(N984Kfs*10) | Inherited from parents | P | Renal insufficiency,Hematuria,Polyhydramnios,Premature birth,Pancytopenia,Abnormality of coagulation,Hyperuricemia,Hepatomegaly |
| 00081058 | ME | u | unknown | No | Yes | VRK1 | NM_003384.2 | Pontocerebellar hypoplasia type 1A(OMIM:607596) | AR | hom | Nonsense | c.1072C>T | p.(R358*) | Inherited from parents | P | Sloping forehead,Talipes equinovarus,Abnormality of the cerebral white matter,Abnormal cortical gyration,Cortical dysplasia,Dilation of lateral ventricles,Hypoplasia of the frontal lobes,Congenital microcephaly,Brain atrophy,Colpocephaly |
| 00081059 | ME | u | unknown | Yes | Yes | DGUOK | NM_080916.1 | m DNA depletion syndrome 3 (hepatocerebral type)(OMIM:251880) | AR | hom | Missense | c.797T>G | p.(L266R) | Inherited from parents | P | Microcephaly,Jaundice,Cholestasis,Hepatomegaly,Short stature,Decreased body weight |
| 00081060 | ME | u | unknown | No | Yes | ADK | NM_006721.3 | Hypermethioninemia due to adenosine kinase deficiency(OMIM:614300) | AR | hom | Nonsense | c.829C>T | p.(R277*) | Inherited from parents | LP | Abnormality of the kidney,Abnormality of head or neck,Hyperinsulinemia,Global developmental delay,Motor delay,Abnormality of the corpus callosum,Cholestasis,Hypoglycemia,Hepatomegaly,CNS hypomyelination,Abnormality of circulating cortisol level |
| 00081061 | SA | u | unknown | Yes | No | DARS | NM_001349.3 | Hypomyelination with brainstem and spinal cord involvement and leg spasticity(OMIM:615281) | AR | hom | Missense | c.766A>C | p.(M256L) | Inherited from parents | P | Nystagmus,Intellectual disability,Spasticity,Hyperreflexia,Developmental regression,Loss of ability to walk,Cherry red spot of the macula,Severe global developmental delay,Abnormal myelination |
| 00081062 | ME | m | 1.1 | Yes | No | EPCAM | NM_002354.2 | Diarrhea 5, with tufting enteropathy, congenital(OMIM:613217) | AR | hom | Splicing | c.556-14A>G | p.? | Inherited from parents | P | Failure to thrive,Vomiting,Chronic diarrhea,Elevated hepatic transaminases,Abnormality of ion homeostasis,Abnormality of the coagulation cascade,Decreased body weight |
| 00081063 | ME | f | 1.0 | No | No | CDKL5 | NM_003159.2 | Epileptic encephalopathy, early infantile, 2(OMIM:300672) | X-Linked | het | Missense | c.119C>A | p.(A40E) | De novo | LP | Seizures,Muscular hypotonia,Global developmental delay,Reduced tendon reflexes |
| 00081064 | ME | m | 16.1 | Yes | Yes | ADCK3 | NM_020247.4 | Coenzyme Q10 deficiency, primary, 4(OMIM:612016) | AR | hom | Missense | c.815G>C | p.(G272A) | Inherited from parents | LP | Delayed speech and language development,Intellectual disability,Seizures,Spasticity,Motor delay,Cerebellar atrophy,Stroke,Hemiplegia/hemiparesis,Brain atrophy |
| 00081065 | ME | u | unknown | No | No | STXBP2 | NM_001272034.1 | Hemophagocytic lymphohistiocytosis, familial, 5(OMIM:613101) | AR | hom | Splicing | c.1485+1G>A | p.? | Inherited from parents | LP | Muscular hypotonia,Hepatosplenomegaly,Neutropenia,Pancytopenia,Anemia,Hepatomegaly,Lactic acidosis |
| 00081066 | Eu | f | 22.7 | No | No | SLC7A7 | NM_001126106.2 | Lysinuric protein intolerance(OMIM:222700) | AR | hom | Splicing | c.895-2A>T | p.? | Inherited from parents | P | Osteoporosis,Thrombocytopenia,Pancytopenia,Leukopenia,Anemia,Hypertriglyceridemia,Increased serum ferritin,Short stature,Abnormality of interleukin secretion,Hemophagocytosis |
| 00081067 | ME | m | 15.9 | Yes | Yes | NDUFV1 | NM_007103.3 | m complex I deficiency(OMIM:252010) | AR | hom | Missense | c.1268C>T | p.(T423M) | Inherited from parents | P | Spasticity,Abnormality of the corpus callosum,Encephalopathy,Dystonia,Hyperreflexia,Brisk reflexes,Failure to thrive,Abnormality of the hair,Abnormal facial shape,Abnormality of the basal ganglia,Cerebellar malformation,Scoliosis,Multiple joint contractures,Hyperalaninemia,Increased serum pyruvate,Elevated brain lactate level by MRS,Abnormality of the substantia nigra |
| 00081068 | ME | m | 30.9 | Yes | N.i | MGME1 | NM_001310338.1 | m DNA depletion syndrome 11(OMIM:615084) | AR | hom | Frameshift | c.634_635del | p.(D212*) | N.i | LP | Bilateral ptosis,Dilated cardiomyopathy,Abnormality of lipid metabolism |
| 00081069 | ME | m | 1.2 | Yes | Yes | SCN1B | NM_001037.4 | Dravet syndrome(PMID:19710327) | AR | hom | Splicing | c.449-2A>G | p.? | Inherited from parents | LP | Global developmental delay,Hyperreflexia,Generalized myoclonic seizures,Muscular hypotonia of the trunk,Feeding difficulties,Epileptic encephalopathy |
| 00081070 | ME | m | 5.7 | Yes | No | SLC37A4 | NM_001164278.1 | Glycogen storage disease Ib(OMIM:232220) | AR | hom | Missense | c.898C>T | p.(R300C) | Inherited from parents | P | Epistaxis,Hepatic steatosis,Macrovesicular hepatic steatosis,Microvesicular hepatic steatosis,Anemia,Diarrhea,Chronic diarrhea,Hepatomegaly,Recurrent upper respiratory tract infections,Elevated hepatic transaminases,Spontaneous, recurrent epistaxis,Elevated serum transaminases during infections,White hair,Abnormal renal morphology |
| 00081071 | SA | m | 1.8 | No | No | TGFB2 | NM_001135599.2 | Loeys-Dietz syndrome 4(OMIM:614816) | AD | het | Missense | c.989G>C | p.(R330P) | De novo | LP | Tall stature,Dolichocephaly,Epicanthus,Hypoplasia of the maxilla,Micrognathia,Low-set ears,Proptosis,Delayed speech and language development,Motor delay,Craniosynostosis,Aortic dilatation,Pes planus,Abnormal facial shape,Aortic root dilatation,High, narrow palate,Sagittal craniosynostosis,Dilatation of the ascending aorta,Prominent metopic ridge,Prominent forehead,Camptodactyly,Abnormality of the thoracic spine |
| 00081072 | ME | m | 5.0 | Yes | No | GJB2 | NM_004004.5 | Keratitis-ichthyosis-deafness syndrome(OMIM:148210) | AD | het | Missense | c.32G>A | p.(G11E) | De novo | LP | Hearing impairment,Sensorineural hearing impairment,Axillary freckling,Respiratory distress,Chronic mucocutaneous candidiasis,Short stature,Decreased body weight,Erythematous papule,Maculopapular exanthema |
| 00081073 | ME | f | 11.8 | Yes | Yes | CLN6 | NM_017882.2 | Ceroid lipofuscinosis, neuronal, 6(OMIM:601780) | AR | hom | Missense | c.662A>C | p.(Y221S) | Inherited from parents | LP | Dementia,Delayed speech and language development,Intellectual disability,Seizures,Ataxia,Muscular hypotonia,Spasticity,Motor delay,Hyperreflexia,Generalized seizures,Developmental regression,Progressive psychomotor deterioration |
| 00081074 | ME | m | 2.0 | No | No | KRAS | NM_033360.2 | RAS-associated autoimmune leukoproliferative disorder(OMIM:614470) | AD | het | Missense | c.182A>C | p.(Q61P) | De Novo | LP | Smooth philtrum,Low-set ears,Abnormality of eye movement,Dry skin,Failure to thrive,Premature birth,Splenomegaly,Thrombocytopenia,Pancytopenia,Anemia,Leukocytosis,Diarrhea,Generalized tonic-clonic seizures,Hepatomegaly,Frequent falls,Decreased body weight,Depressed nasal bridge,Loss of consciousness,Ichthyosis,Bilateral cryptorchidism,Generalized lymphadenopathy,Cephalohematoma,Telangiectasia of the skin |
| 00081075 | Eu | f | 1.6 | No | No | KAT6A | NM_006766.4 | Mental retardation, autosomal dominant 32(OMIM:616268) | AD | het | Frameshift | c.3655del | p.(L1219Yfs*75) | De Novo | LP | Delayed speech and language development,Poor eye contact,Intellectual disability,Muscular hypotonia,Motor delay,Abnormal facial shape,Mild microcephaly |
| 00081076 | ME | m | unknown | N.i | N.i | ADAT3 | NM_138422.2 | Mental retardation, autosomal recessive 36(OMIM:615286) | AR | hom | Missense | c.430G>A | p.(V144M) | Inherited from parents | P | Triangular face,Macrotia,Deeply set eye,Synophrys,Autistic behavior,Delayed speech and language development,Muscular hypotonia,Global developmental delay,Abnormal facial shape,Frontal bossing,Prolonged neonatal jaundice,Cerebral hypomyelination,Narrow nasal tip |
| 00081077 | ME | f | 18.5 | Yes | Yes | KCNQ1 | NM_000218.2 | Jervell and Lange-Nielsen syndrome(OMIM:220400) | AR | hom | Splicing | c.387-5T>A | p.? | Inherited from father& | P | Tachycardia,Prolonged QT interval,Vertigo |
| 00081078 | Am | m | 51.9 | No | No | NEFH | NM_021076.3 | Amyotrophic lateral sclerosis, susceptibility to(OMIM:105400) | AD | het | Frameshift | c.3023dup | p.(E1009Rfs*54) | Inherited from mother* | LP | Muscle weakness,Back pain,Difficulty climbing stairs,Lower limb muscle weakness |
| 00081079 | ME | m | 19.4 | Yes | Yes | SPG11 | NM_025137.3 | Spastic paraplegia 11, autosomal recessive(OMIM:604360) | AR | hom | Nonsense | c.3121C>T | p.(R1041*) | Inherited from mother& | P | Ataxia,Spasticity,Hypertonia,Hyperreflexia,Gait ataxia |
| 00081080 | ME | u | prenatal | Yes | N.i | CC2D2A | NM_001080522.2 | COACH syndrome(OMIM:216360) | AR | hom | Frameshift | c.650del | p.(G217Efs*41) | N.i | LP | Abnormality of the kidney,Occipital encephalocele,Polydactyly,Abnormality of brain morphology |
| 00081081 | Eu | f(46,XY) | 31.9 | No | Yes | AR | NM_000044.3 | Androgen insensitivity(OMIM:300068) | X-linked | hemi | Nonsense | c.238C>T | p.(Q80*) | Inherited from mother | P | Abnormality of the genital system,Primary amenorrhea,Infertility,Elevated luteinizing hormone,Abnormality of the uterine cervix,Increased serum testosterone level |
| 00081082 | ME | f | 4.6 | N.i | Yes | SUOX | NM_000456.2 | Sulfite oxidase deficiency(OMIM:272300) | AR | hom | Frameshift | c.520del | p.(D174Tfs*13) | Inherited from mother& | P | Microcephaly,Seizures,Global developmental delay,Failure to thrive,Cerebellar agenesis |
| 00081083 | ME | f | 1.3 | N.i | No | ADK | NM_006721.3 | Hypermethioninemia due to adenosine kinase deficiency(OMIM:614300) | AR | hom | Frameshift | c.813dup | p.(N272Efs*16) | Inherited from mother& | LP | Muscular hypotonia,Hip dysplasia,Hypermethioninemia,Central hypotonia |
| 00081084 | SA | m | unknown | Yes | Yes | CDH23 | NM_022124.5 | Deafness, autosomal recessive 12(OMIM:601386) | AR | hom | Missense | c.778G>A | p.(A260T) | Inherited from father& | P | Hearing impairment |
| 00081085 | SA | f | 2.3 | No | Yes | CDKL5 | NM_003159.2 | Epileptic encephalopathy, early infantile, 2(OMIM:300672) | X-linked | het | Nonsense | c.858C>A | p.(Y286*) | De Novo | LP | Abnormality of periauricular region,Cafe-au-lait spot,Seizures,Global developmental delay,Muscle weakness,Brain atrophy,Abnormality of movement |
| 00081086 | ME | f | 4.5 | Yes | No | SCN2A | NM_021007.2 | Epileptic encephalopathy, early infantile, 11(OMIM:613721) | AD | het | Nonsense | c.3827G>A | p.(W1276*) | De Novo | LP | Delayed speech and language development,Intellectual disability,Muscular hypotonia,Global developmental delay,Motor delay,Generalized seizures |
| 00081087 | ME | m | 18.5 | Yes | Yes | FOLR1 | NM_016724.2 | Neurodegeneration due to cerebral folate transport deficiency(OMIM:613068) | AR | hom | Missense | c.398C>A | p.(P133H) | Inherited from parents | P | Delayed speech and language development,Seizures,Global developmental delay,Cerebellar atrophy,Cerebral atrophy,Basal ganglia calcification,Neurodegeneration,EEG abnormality,Abnormality of the periventricular white matter,Intracranial cystic lesion,Epileptiform EEG discharges,Brain atrophy |
| 00081088 | ME | m | 15.2 | Yes | No | SNX14 | NM_153816.3 | Spinocerebellar ataxia, autosomal recessive 20(OMIM:616354) | AR | hom | Splicing | c.1894+1G>T | p.? | Inherited from parents | P | Delayed speech and language development,Intellectual disability,Ataxia,Cerebellar cortical atrophy |
| 00081089 | ME | u | unknown | Yes | Yes | ALG3 | NM_005787.5 | Congenital disorder of glycosylation, type Id(OMIM:601110) | AR | hom | Missense | c.512G>A | p.(R171Q) | Inherited from parents | P | Micropenis,Enlarged kidneys,Renal dysplasia,Narrow mouth,Microcephaly,Abnormality of the thorax,Short ribs,Congenital diaphragmatic hernia,Platyspondyly,Abnormality of the skull,Cerebellar vermis hypoplasia,Cerebellar hypoplasia,Lissencephaly,Hepatic cysts,Intrauterine growth retardation,Abnormality of the metatarsal bones,Hypoglycemia,Abnormal facial shape,Hepatomegaly,Cavum septum pellucidum,Abnormality of the cerebral cortex,Thoracolumbar scoliosis,Rhizomelic arm shortening,Opacification of the corneal stroma,Long toe,Wide cranial sutures,Increased bone mineral density,Medullary nephrocalcinosis,Cortical nephrocalcinosis,Abnormality of the thoracic spine,Long fingers,Septo-optic dysplasia |
| 00081090 | ME | m | 5.9 | Yes | N.i | MGP | NM_001190839.1 | Keutel syndrome(OMIM:245150) | AR | hom | Splicing | c.169+1G>A | p.? | N.i | P | Cryptorchidism,Micropenis,Microcephaly,Coarse facial features,Cerebellar hypoplasia,obsolete Malformation of the heart and great vessels,Dilation of lateral ventricles,Periventricular leukomalacia,Posterior fossa cyst |
| 00081091 | ME | m | 1.3 | Yes | No | SETBP1 | NM_015559.2 | Schinzel-Giedion midface retraction syndrome (OMIM:269150) | AD | het | Missense | c.2602G>A | p.(D868N) | De Novo | P | High palate,Microcephaly,Low-set ears,Prominent nose,Proptosis,Delayed speech and language development,Spasticity,Motor delay,Abnormal facial shape,Generalized seizures,Developmental regression |
| 00081092 | ME | f | 12.7 | Yes | N.i | NOTCH2 | NM_024408.3 | Alagille syndrome 2(OMIM:610205) | AD | het | Nonsense | c.6007C>T | p.(R2003*) | De novo | P | Microcephaly,Strabismus,Congenital diaphragmatic hernia,Osteopenia,Muscular hypotonia,Congenital hip dislocation,Cholestasis,Abnormal facial shape,Mild global developmental delay |
| 00081093 | ME | f | 0.7 | Yes | Yes | RAB3GAP1 | NM_001172435.1 | Warburg Micro syndrome 1 (OMIM:600118) | AR | hom | Splicing | c.899+1G>A | p.? | Inherited from parents | P | Triangular face,Cataract,Motor delay,Agenesis of corpus callosum,Failure to thrive,Abnormal facial shape,Polydactyly |
| 00081094 | ME | f | 0.1 | Yes | No | COL6A2 | NM_001849.3 | Ullrich congenital muscular dystrophy 1(OMIM:254090) | AR | hom | Splicing | c.2422+1G>A | p.? | Inherited from parents | LP | Dolichocephaly,Micrognathia,Low-set ears,Short neck,Telecanthus,Arachnodactyly,Intrauterine growth retardation,Abnormal facial shape,Hip dislocation,Multiple joint contractures,Methylmalonic acidemia,Decreased adenosylcobalamin,Limited elbow flexion,Limited knee flexion,Feeding difficulties,Long fingers |
| 00081095 | ME | f | 2.0 | No | No | IL12B | NM_002187.2 | Immunodeficiency 29,mycobacteriosis(OMIM:614890) | AR | hom | Frameshift | c.320dup | p.(E108Gfs*8) | Inherited from parents | P | Hepatomegaly,Lymphadenitis,Generalized lymphadenopathy |
| 00081096 | ME | f | 5.8 | Yes | Yes | HPS3 | NM_032383.3 | Hermansky-Pudlak syndrome 3(OMIM:614072) | AR | hom | Frameshift | c.1153_1160del | p.(V385Kfs*2) | Inherited from parents | LP | Sensorineural hearing impairment,Visual impairment,Hypermetropia,Nystagmus,Delayed speech and language development,Muscular hypotonia,Global developmental delay,Motor delay,Attention deficit hyperactivity disorder |
| 00081097 | ME | m | 3.0 | N.i | N.i | PRDM12 | NM_021619.2 | Neuropathy, hereditary sensory and autonomic, type VIII(OMIM:616488) | AR | hom | Frameshift | c.172dup | p.(S58Kfs*85) | Inherited from parents | P | Narrow mouth,Deeply set eye,Downslanted palpebral fissures,Nystagmus,Delayed speech and language development,Ataxia,Short nose,Pain insensitivity,Mild global developmental delay |
| 00081098 | ME | f | 5.8 | No | N.i | CCDC103 | NM_213607.2 | Ciliary dyskinesia, primary, 17(OMIM:614679) | AR | hom | Missense | c.461A>C | p.(H154P) | Inherited from parents | P | Delayed speech and language development,Global developmental delay,Motor delay,Heterotopia,obsolete Malformation of the heart and great vessels,Bronchitis,Abnormality of brain morphology |
| 00081099 | ME | f | 3.8 | N.i | N.i | PKD1 | NM_001009944.2 | Polycystic kidney disease, adult type I(OMIM:173900) | AD | het | Frameshift | c.7174del | p.(R2392Afs*228) | De Novo | LP | Multicystic kidney dysplasia,Vesicoureteral reflux,Enlarged kidneys,Polycystic kidney dysplasia |
| Description of columns: #: case number; Geographic origin: the world area of the probe with ME for Middle East, Eu for Europe, Am for North and South America, SA for South Asia, and one probe from Oceania; Sex: female (f), male (m) or unknown (u); consanguinity: are the parents related: yes, no, or n.i (n.i.); positive family history: any other affected members other than the index: yes, no or n.i (n.i.); gene: gene id; transcript: number of transcript (important for the c and p positions); OMIM description and number or PubMed ID when relevant; Inheritance: autosomal recessive (AR), autosomal dominant (AD), mitochondrial (M), or X linked (X); Zygosity: homozygous variant (hom), heterozygous (het), heterplasmic (het-pl), compound heterozygous (comp het), or hemizygous (hemi); coding effect: nonsense, missense, etc.; cDNA change: the change on the coding DNA level; AA change: the change on the protein level (when applicable); family segregation: inherited from parents (when homozygous), or from one parent (when heterozygous), or de novo; significance: pathogenic (P) or likely pathogenic (LP) according to ACMG guidelines; HPO terms: translation of given clinical information to the HPO system. | | | | | | | | | | | | | | | | |
